# IsoSel: Protein Isoform Selector for phylogenetic reconstructions

**DOI:** 10.1371/journal.pone.0174250

**Published:** 2017-03-21

**Authors:** Héloïse Philippon, Alexia Souvane, Céline Brochier-Armanet, Guy Perrière

**Affiliations:** Univ. Lyon, Université Claude Bernard Lyon 1, CNRS, Laboratoire de Biométrie et Biologie Evolutive, UMR 5558, F-69622, Villeurbanne, France; Universite Paris-Sud, FRANCE

## Abstract

The reliability of molecular phylogenies is strongly dependent on the quality of the assembled datasets. In the case of eukaryotes, the selection of only one protein isoform per genomic locus is mandatory to avoid biases linked to redundancy. Here, we present IsoSel, a tool devoted to the selection of alternative isoforms in the context of phylogenetic reconstruction. It provides a better alternative to the widely used approach consisting in the selection of the longest isoforms and it performs better than Guidance, its only available counterpart. IsoSel is publicly available at http://doua.prabi.fr/software/isosel.

## Introduction

The alternative splicing, a process by which a single coding gene may lead to different transcripts and thus to different protein isoforms, is common in Eukaryotes. For instance, about 20% of plant genes [1] and 90% of human genes [2] undergo alternative splicing. In molecular phylogeny, the construction of homologous sequences datasets—usually performed by similarity-based procedures—does not allow distinguishing among the various isoforms and all of them are gathered during the process. However, most of the time only one isoform is kept for phylogenetic analyses, because they carry redundant information. Furthermore, due to the fact that some exons are present in some isoforms and absent in others, aligning them frequently leads to the introduction of many gaps in Multiple Sequence Alignments (MSAs) [3]. Finally, trimming programs like Gblocks [4] or BMGE [5] select alignment regions based on their conservation level. So, introducing many isoforms will lead to the overestimation of conservation rates, a same residue being artefactually represented many times in the MSA.

It is therefore absolutely necessary to select a unique sequence per genomic locus before reconstructing a phylogeny. Although manual selection provides usually the best results, this becomes tedious when the number of sequences and/or of homologous families is large. Two simple automated approaches are therefore commonly used: the random selection of one isoform [6, 7] or the selection of the longest isoform [8, 9]. But there is no conceptual justification for the former and the latter usually leads to the introduction of many gaps in the alignments. Presently, only one software dedicated to isoform selection is available: PALO [10]. It selects the combination of isoforms that are most similar in length. However, PALO only works on sequences from the Ensembl database and its unique selection criterion is based on sequence length.

In that context, we developed IsoSel (Isoform Selector), a tool designed to the selection of protein isoforms specifically designed for phylogenetic reconstruction. IsoSel is based on the same approach as the one used by Guidance [11], a tool devoted to the assessment of protein MSAs reliability. The two main differences between IsoSel and Guidance are the availability of a broader range of amino acids substitution models in IsoSel and the introduction of different options allowing to penalize short and long sequences. IsoSel can also provide an output file giving the corresponding selected isoform for each genomic locus. Most frequently, the sequences selected by IsoSel allows to build phylogenetic trees that are better than those obtained with the sequences selected by Guidance, this when considering a tree length criterion and the number of Duplication-Loss (DL) events inferred in a tree reconciliation. Lastly, IsoSel is a standalone program that does not require the availability of interpreted languages such as Perl and Ruby. It is therefore easier to install and to use.

## Materials and methods

### Algorithm

**Fig 1:**
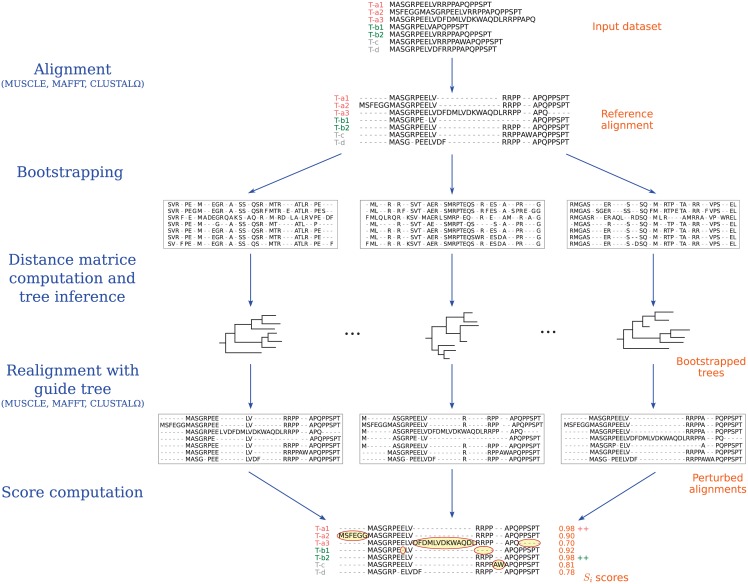
IsoSel workflow. Schematic representation of the different steps performed during an IsoSel run. T-x represent alternatives isoforms generated by a same gene x. In this example, isoforms a1 and b2 are selected for the genes a and b, respectively.

The first step of an IsoSel run consists in the alignment of an input protein dataset, using either CLUSTALO [12], MAFFT [13] or MUSCLE [14], to generate what we call a reference alignment. We selected those three programs because they allow to input a user-provided guide tree when building a MSA, which is required during the second step of the algorithm (Fig 1).

The second step is the generation of a set of perturbed alignments through a bootstrap approach. Let *a* be the reference alignment obtained during step 1, *ℓ* the length of this alignment and *n* the number of bootstrap replicates set by the user. First, *n* alignment replicates are generated by the standard bootstrap procedure (*i.e*., random sampling with replacement of *ℓ* sites among *a*). For each bootstrap replicate, a distance matrix is then computed by IsoSel, this using one of the amino acid substitution models implemented in the program: Poisson (with or without Gamma correction), PAM (or its Kimura approximation), JTT (or its Gamma-corrected Poisson approximation), BLOSUM62, WAG or LG. From this distance matrix, the BioNJ [15] algorithm is used to infer a tree. Finally, the input dataset is realigned using this tree as guide tree, this with the same MSA program as the one used the first step. The *n* resulting realignments of *a* represent the perturbed alignments.

**Fig 2:**
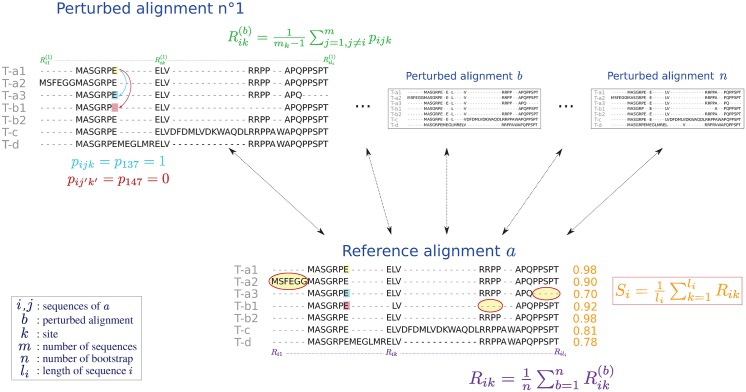
SP score computation. Example of score computation for four genes (a, b, c and d) producing three (a), two (b) and no (c and d) alternative isoforms.

The third and final step is the computation of the Sum-of-Pairs (SP) score [16] using the perturbed alignments. As described in the original publication, the SP score is used as a comparison metric between two MSAs (Fig 2).

Let *m* be the number of sequences of the input dataset and *b* (1 ≤ *b* ≤ *n*) one of the *n* perturbed alignments generated during the second step. For each sequence *i* (1 ≤ *i* ≤ *m*) of length *l*_*i*_ from *b*, the pair score of the amino acid at position *k* (1 ≤ *k* ≤ *l*_*i*_) is computed as:

$$R_{ik}^{\left(b\right)}=\frac{1}{m_{k}-1}\;\sum_{j=1,j\neq i}^{m_{k}}p_{ijk}$$ (1)

where *p*_*ijk*_ is equal to 1 or 0 whether or not the amino acid at position *k* of sequence *i* is facing the same amino acid at the position *k*′ (1 ≤ *k*′ ≤ *l*_*j*_) of sequence *j* in the reference and perturbed alignment (Fig 2). Also, *m*_*k*_ is the number of sequences having a residue (*i.e*., not a gap) at this position in *a*. If there no other residue at this position (*i.e*., if *m*_*k*_ = 1), $R_{ik}^{\left(b\right)}$ is set to 1.

From Eq (1) it is possible to compute the average residue score over all bootstrap replicates as:

$$R_{ik}=\frac{1}{n}\;\sum_{b=1}^{n}R_{ik}^{\left(b\right)}$$ (2)

Finally, the SP score *S*_*i*_ for sequence *i* is calculated by averaging the residues scores:

$$S_{i}=\frac{1}{l_{i}}\;\sum_{k=1}^{l_{i}}R_{ik}$$ (3)

By construction, 0 ≤ *S*_*i*_ ≤ 1. The higher its value, the better the alignment of the isoform with the other sequences. For each genomic locus, the isoform with the best score is kept.

### Special options

IsoSel comes with a lot of available options and parameters (for the substitution models, the Gamma correction, the alignment programs, etc.) and we will not list them all. But some of those options are of special interest as they can allow to improve greatly the results depending on the dataset contents. Below is a short description of those key options and in which context they should be used.

#### The -gap option

The standard SP score described in the previous section does not take into account the number of gaps present at a given site of *a*. For that purpose, we have implemented an option (-gap) which penalizes the sequences introducing gaps in the reference alignment. With this option, *p*_*ijk*_ is weighted by the number of gaps present at position *k* in *a*:

$$R_{ik}^{\left(b\right)}=\frac{1}{m_{k}-1}\;\sum_{j=1,j\neq i}^{m_{k}}\frac{p_{ijk}}{m_{g}}$$ (4)

where *m*_*g*_ = *m* − *m*_*k*_ is the number of gaps at this position in *a*. If there is no gap, then *m*_*g*_ = 1. Here, *p*_*ijk*_ is the same as before excepted that it is fixed at −1 if there is no other residue at this position in the reference alignment. By construction, *S*_*i*_ values are computed using the same equation as before but they are much smaller. Nevertheless the alternative transcript selected is always the one with the highest score. This option is suited for datasets containing alternative isoforms resulting from intron retention or exon skipping events that are restricted to a reduced set of taxonomic groups.

#### The -short option

If the option -gap penalizes too long isoforms, the option -short was designed to penalize short isoforms, even if they are well aligned. In this case, *S*_*i*_ is no longer calculated by dividing the sum of residue scores by the length of the sequence, but by the length of the reference alignment:

$$S_{i}=\frac{1}{\ell }\;\sum_{k=1}^{l_{i}}R_{ik}$$ (5)

As for the -gap option, the *S*_*i*_ values obtained are, by construction, smaller. This option is suited for datasets with isoforms corresponding to partial sequences or when a very short isoform is specific to a given taxonomic group.

#### The -DS and -WOT options

The bootstrap approach, with the computation of a set of perturbed alignments, is very time consuming when the number and/or the length of the sequences increase. To allow a faster approach, the -DS option (for “Distance Scores”) approximates the SP score by the mean of observed divergence (*p*-distance) between sequence *i* and the other input dataset sequences. Unlike the other scoring schemes, there is no bootstrap resampling procedure and the isoform selected is the one with the smallest distance (*i.e*., the sequence selected is the one that it is the more similar to the others).

In order to take into account the difference of lengths between isoforms, the -DS option uses a modified *p*-distance in which a gap is considered as a supplementary character state. In that way, considering two isoforms that only differs by an exon skipping event, their modified *p*-distance will not be near to one as it is with the standard *p*-distance. A bias towards the systematic selection of smaller isoform is thus avoided.

Let *d*_*ij*_ be the modified *p*-distance between sequences *i* and *j* (1 ≤ *i*, *j* ≤ *m*). The score *S*_*i*_ corresponding to the sequence *i* is then computed as:

$$S_{i}=\frac{1}{m_{s}}\;\sum_{i\in \Omega ,i\neq j}d_{ij}$$ (6)

where Ω corresponds to the subset of sequences without isoform and *m*_*s*_ its cardinal. The higher *S*_*i*_, the more distant to the other sequences of the input dataset is the sequence *i*.

In combination to the -DS option, the -WOT option (for “With Other Transcripts”) computes the mean of distances on the entire input dataset. Then Eq (6) becomes:

$$S_{i}=\frac{1}{m_{s}}\;\sum_{i=1,i\neq j}^{m}d_{ij}$$ (7)

This option is suited for datasets containing a majority of homologous genes generating alternative isoforms.

#### The -auto option

Best options for IsoSel are dataset dependent, therefore we have implemented an automated mode (-auto) allowing to estimate them. Under this mode, if the reference alignment has > 35% of sites containing > 80% gaps, the *S*_*i*_ scores are computed with the -gap option. If there are > 600 sequences in the input dataset or if the alignment length is > 10000 AA, then the option -DS is selected. In all other cases, the default parameters are used.

## Input and output

**Fig 3:**
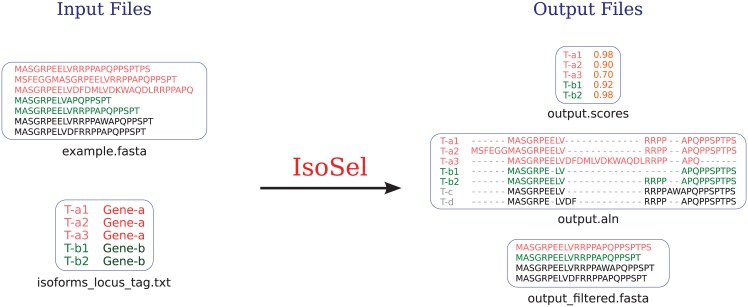
Input and output files. IsoSel minimal input requirement is an unaligned protein sequence dataset in Fasta format (example.fasta). The two output files generated contain the alignment (output.aln) and the sequences scores (output.scores or output.DistanceScore if the -DS option is used). Optionally, the user can provide a file containing the genomic origin of the input sequences (isoforms_locus_tag.txt). In this case, an additional file containing, for each locus, the sequence having the highest score is created (output_filtered.fasta).

IsoSel minimal input requirement is an unaligned set of protein sequences in Fasta format. The output is a text file containing the scores for each input sequence (Fig 3). Optionally, the user can provide a file in which the information on transcripts locus tag is given. In this case, IsoSel will also create a file in Fasta format that will contain the filtered dataset (*i.e*., in which only the isoform having the best score for a given gene is kept).

An example dataset containing two files is included in the program distribution. The first one, named example.fasta, contains 58 homologs of the human AKTS1 protein taken from Ensembl and from a local database of complete eukaryotic proteomes. Among those 59 sequences, 26 correspond to alternative isoforms whose genomic origin is indicated in the second file, named isoforms_locus_tag.txt. The first column of this second file corresponds to the sequence names (the same as those in the example.fasta file) and the second column to an identifier allowing associating a set of isoforms to a given gene. For example, the three lines below:

>ENSP00000375710|Homo_sapiens ENSG00000204673
>ENSP00000375711|Homo_sapiens ENSG00000204673
>ENSP00000375706|Homo_sapiens ENSG00000204673

allows to specify that sequences ENSP00000375710, ENSP00000375711 and ENSP00000375706 are isoforms that originate from a single gene, identified as ENSG00000204673.

### Datasets for program testing

We randomly sampled 200 human proteins among the 20201 available in UniProtKB release 2016_05. For each sequence, we searched for its homologs into two collections using BLASTP [17], this with a similarity threshold set at *E* ≤ 10^−30^. The first collection corresponded to a subset of Ensembl release 80 containing 32 species while the second was made of 84 complete eukaryotic proteomes taken from GenBank release 70 (S1 and S2 Tables, respectively). Among the 200 human proteins used as seed for BLASTP, 12 corresponded to ORFans and 32 returned less than 20 homologous genes. Moreover, two BLASTP runs contained only one gene with two alternative isoforms. All those sets were discarded and the remaining 154 were used to test the performances of IsoSel relatively to the other possible strategies (S3 Table).

**Fig 4:**
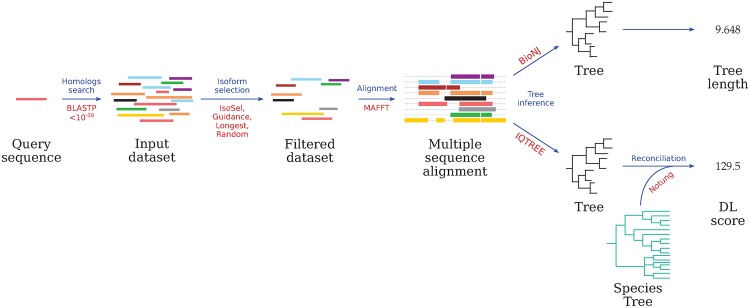
Workflow used for testing IsoSel performances. For a given human protein from UniProtKB, a BLASTP search is performed. The alternative isoforms detected for each set of homologs are then selected using either the longest isoform, a random choice, Guidance or IsoSel. Then the sets are aligned and the corresponding gene trees are inferred by BioNJ and IQ-TREE for computing tree lengths and DL scores, respectively. For each step, algorithms used are indicated in red.

The complete workflow summarizing the testing procedure is shown in Fig 4. For each of the 154 sets of homologs, we ran IsoSel with the different available options and 30 bootstraps replicates. We compared the results returned by IsoSel to selections obtained with: i) the longest isoforms; ii) the random choice of an isoform for each genomic locus; and iii) the scores obtained using Guidance 2.0 [18] with 30 bootstrap replicates and default parameters.

The resulting filtered datasets, containing only one sequence per genomic locus, were aligned using MAFFT (automatic algorithm selection and a maximum of 20 iterations). From each MSA, two trees were inferred to compute the tree length and the DL score. The first tree was inferred by SeaView [19] using the *p*-distance and the BioNJ algorithm. Then, the sum of the branch lengths was computed and all tree lengths obtained are listed in S4 Table. For the second gene tree inference, we run BMGE to select conserved blocks of the MSA (BLOSUM30 substitution matrix, 40% of gap allowed and a minimal block length of three amino acids). Then, the selection of evolutionary models (Bayesian Information Criterion) and the tree inference was computed by maximum likelihood using IQ-TREE [20] with default parameters and 1000 replicates for the Shimodaira-Hasegawa-like approximate likelihood ratio test (SH-aLRT). Finally, the resulting gene tree was rooted and reconciled with a reference tree of eukaryotic species [21, 22] (S1 Fig) using Notung [23]. All the DL scores obtained are listed in S5 Table.

Among the 154 sets, we used the one built with the human protein WDR18 (UniProtKB accession number Q9BV38) as a case study. For this sequence, the BLASTP search in the two collections led to a set of 73 homologous protein sequences. Among them, fourteen (19.2%) resulted from alternative splicing events (S3 Table). Selection of isoforms from this set was performed by the longest sequence criterion and by IsoSel (default parameters). For both selections (containing 63 sequences) we applied the methodology described above for computing maximum likelihood trees. The two filtered alignments contained a total of 349 and 354 conserved sites, respectively. In both cases, the evolutionary model selected by IQ-TREE was LG+F+Γ_4_ [24]. The resulting trees were then formatted using TreeGraph2 [25].

## Results

### Tree length criterion

**Fig 5:**
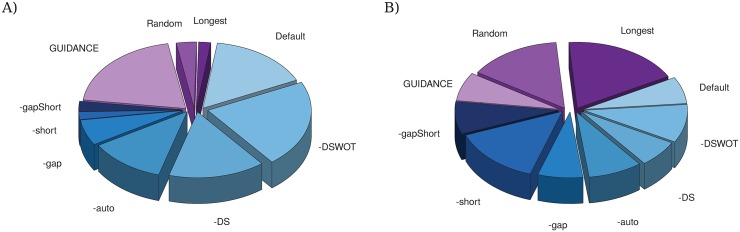
Tree lengths and DL scores distributions. Charts are proportional to the number of: A) the shortest trees; and B) the trees with the lower DL score obtained with the different options and programs. Charts in shades of blue correspond to the different IsoSel options.

There is presently no gold standard to evaluate the quality of a MSA relatively to a phylogenetic criterion. However, Rzhetsky and Nei [26], stated that the tree with the smallest sum of branches length is most likely to be the true one. We therefore used this criterion to roughly estimate the quality of the MSAs. We compared the length of the trees generated with the IsoSel filtered datasets to those obtained by keeping a random isoform, the longest isoform or by a selection with Guidance. For that purpose, we used the 154 sets of eukaryotic protein families obtained through the procedure described above. For more than 76% of the test datasets, the use of IsoSel led to a shorter tree than the one obtained using the other approaches (S4 Table and Fig 5A).

The longest and the random isoforms selections led to the smallest trees in only three and five cases, respectively. Moreover, for 129 and 127 of the 154 datasets (corresponding to 83.7% and 82.4% of the total), using IsoSel with the -auto option led to shorter trees than the random and the longest isoform selections, respectively. A Wilcoxon paired test showed that these results are highly significant (both *P* < 2.2 × 10^−16^). In 119 cases (78.8%), one of the IsoSel options led to a shorter tree than the Guidance selection, which is also highly significant (*P* < 5.27 × 10^−12^).

### DL score criterion

Another criterion of gene tree quality is its congruence to a reference species tree, especially in the case of eukaryotic species where horizontal gene transfers are rare events. We therefore performed a tree reconciliation for each of our datasets using Notung. The DL score provided by this program is proportional to the minimum number of duplications and losses needed to reconcile a gene tree with a species tree. Therefore, the lower the score is, the closer the gene tree is to the species tree.

For 122 datasets (corresponding to 79.2% of the total), one of the IsoSel options allowed to obtain a tree with an equal (20 cases) or better (102 cases) DL score than the one inferred after the selection of the longest isoform. The comparison with the random selection gave similar results as IsoSel was better in 117 cases and equal in seven (corresponding to 75.9% and 4.5%, respectively). Finally, in 128 cases (84.7%), IsoSel gave better results than the Guidance selection and performed equally in thirteen cases (8.6%). All those results are highly significants (all Wilcoxon paired tests led to *P* < 10^−8^). Globally, for 63.5% of the datasets, the use of IsoSel led to equal or less discordant gene trees than those obtained using the other approaches (Fig 5B).

### WDR18 protein

**Fig 6:**
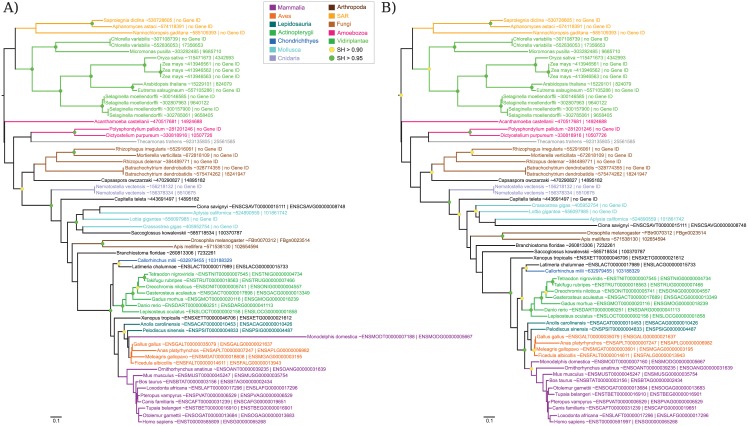
Maximum likelihood trees for WDR18 protein. Isoform selection was done by selecting the longest isoform (A) and by running IsoSel with its default parameters (B). Sequences are colored according to their taxonomic classification. Green and yellow circles correspond to nodes with SH > 0.95 and SH > 0.90, respectively. The scale bar represents the average number of substitutions per site.

The phylogenetic trees obtained using with the longest and the IsoSel (with default parameters) procedures are shown in Fig 6. The selection carried out by IsoSel led to a shorter but more discordant gene tree than the selection using the longest isoforms (S4 and S5 Tables). The selection using the longest sequences led to a phylogenetic tree in which the isoform selected for *Monodelphis domestica* (ENSMODT00000007188) is misplaced, this erroneous placement being probably linked to the long branch generated (Fig 6A). *Saccoglossus kowalevskii* is also incorrectly placed in the selection using the longest sequences. Its position is improved in the IsoSel selection but there is no statistical supported for the placement in both trees. Another consequence of the longest isoforms selection is that the clade grouping mammals other than *M. domestica* is not supported, while it is in the tree obtained with the IsoSel set (SH = 0.92, Fig 6B). Similarly, the clade corresponding to the Amniotes is only supported in the tree obtained with the IsoSel selection (SH = 0.98). On the other hand, according to our reference tree for eukaryotic species (S1 Fig), the human sequence is incorrectly grouped with the elephant and *Xenopus laevis* is misplaced in the tree built with the IsoSel selection.

## Conclusion

IsoSel is a command line software designed for the automatic selection of protein isoforms in the framework of phylogenetic analyses. Based on the SP score, it allows to obtain datasets that are optimized for tree reconstruction. The only other software that can be compared to IsoSel is Guidance but this program presents some limitations. First, it requires the independent installation of a broad range of tools (namely Perl, BioPerl and Ruby) while IsoSel is self-sufficient and is distributed with all the binaries required for its functioning. Then, it can only be run with the JTT substitution model while IsoSel allows the use of all standard site-homogeneous models. On a practical point of view, Guidance is usually slower than IsoSel when multithreading is enabled (data not shown). This point is probably linked to the fact that Guidance was not designed for alternative isoforms selection but rather as a general tool for assessing MSA quality. With this broader purpose, Guidance has to compute many scores in addition to SP, which lower its performances in terms of speed.

Globally, it appears that, compared to the other available approaches, IsoSel allows selecting most frequently the sequences leading to gene trees that are shorter and closer to the species tree. It is thus more suited for phylogenetic reconstructions. IsoSel is implemented in C/C++, is optimized for multithreading and is available under the CeCILL license.

## Supporting information

S1 Table Sizes of the proteomes selected from Ensembl. Number of protein sequences available in Ensembl for each of the 32 selected species.
(PDF)

S2 Table Sizes of the complete eukaryotic proteomes selected from GenBank. Number of protein sequences for each selected eukaryotic complete genomes. The selected species are different from the ones from Ensembl.
(PDF)

S3 Table Characteristics of the 200 test datasets. For each of the randomly selected human protein, the number of homologous sequences detected using BLASTP is indicated in the third column. The fourth and fifth columns give the gene number and percentage of alternative isoforms, respectively. Alignment length and different statistics about the detected homologs are listed in the last columns. ORFans, datasets containing less than 20 homologs or with only one gene generating alternative isoforms are highlighted in light grey, blue and yellow, respectively.
(PDF)

S4 Table Trees length. For each of the 154 used datasets, the tree length obtained with each method is listed. The shortest are highlighted in blue. For three datasets (corresponding to proteins Q6ZN06, P58317 and Q8N8J6), the selection with Guidance failed due to program crash. They are highlighted in light orange.
(PDF)

S5 Table DL scores. For each of the 154 used datasets, the DL score computed by Notung with each isoform selection strategy is listed. The most consistent are highlighted in blue. For three datasets (corresponding to proteins Q6ZN06, P58317 and Q8N8J6), the selection with Guidance failed due to program crash. They are highlighted in light orange.
(PDF)

S1 Fig Reference phylogenetic tree of eukaryotic species used for the gene trees reconciliations. This tree was built according to Lecointre and Le Guyader book [21], the Ensembl reference species tree for the metazoan part [22] and a personal communication from CBA.
(PDF)
