# Supplementary material for: IsoSel: Protein Isoform Selector for phylogenetic reconstructions
Source: PLoS One. 2017 Mar 21;12(3):e0174250. doi: 10.1371/journal.pone.0174250 (PMC5360266; doi:10.1371/journal.pone.0174250)
Supplement: S1 Table — Number of protein sequences available in Ensembl for each of the 32 selected species. (PDF) [file pone.0174250.s001.pdf]

Species	Number of sequences
Anas platyrhynchos	16353
Anolis carolinensis	19177
Bos taurus	22118
Caenorhabditis elegans	30939
Canis familiaris	25160
Ciona intestinalis	17302
Ciona savignyi	20155
Danio rerio	44487
Drosophila melanogaster	30362
Erinaceus europaeus	14605
Ficedula albicollis	15983
Gadus morhua	22100
Gallus gallus	16354
Gasterosteus aculeatus	27576
Homo sapiens	100778
Latimeria chalumnae	23601
Lepisosteus oculatus	22483
Loxodonta africana	25635
Meleagris gallopavo	16494
Monodelphis domestica	22310
Mus musculus	54883
Oreochromis niloticus	26763
Ornithorhynchus anatinus	23584
Otolemur garnettii	19986
Pelodiscus sinensis	20669
Petromyzon marinus	11442
Pteropus vampyrus	17053
Saccharomyces cerevisiae	6692
Takifugu rubripes	47841
Tetraodon nigroviridis	23118
Tupaia belangeri	15475
Xenopus tropicalis	22718
**Total :**	**824196**
