# Supplementary material for: IsoSel: Protein Isoform Selector for phylogenetic reconstructions
Source: PLoS One. 2017 Mar 21;12(3):e0174250. doi: 10.1371/journal.pone.0174250 (PMC5360266; doi:10.1371/journal.pone.0174250)
Supplement: S2 Table — Number of protein sequences for each selected eukaryotic complete genomes. The selected species are different from the ones from Ensembl. (PDF) [file pone.0174250.s002.pdf]

Species	Number of sequences	Species	Number of sequences
Acanthamoeba castellanii	15014	*Lottia gigantea*	23877
Allomyces macrogynus	19446	*Magnaporthe oryzae*	12836
Amphimedon queenslandica	9914	*Micromonas pusilla*	10269
Angomonas deanei	16888	*Monosiga brevicollis*	9203
Aphanomyces astaci	26259	*Mortierella verticillata*	12660
Apis mellifera	21772	*Mucor circinelloides*	12368
Aplysia californica	26005	*Naegleria gruberi*	15711
Arabidopsis thaliana	35378	*Nannochloropsis gaditana*	19601
Aspergillus oryzae	12074	*Nematocida parisii*	5387
Aureococcus anophagefferens	11520	*Nematostella vectensis*	50384
Batrachochytrium dendrobatidis	17425	*Nosema bombycis*	4764
Blumeria graminis	6525	*Oryza sativa*	28553
Branchiostoma floridae	28623	*Oxytricha trifallax*	24578
Callorhinchus milii	28224	*Paramecium tetraurelia*	39578
Candida tropicalis	6254	*Phaeodactylum tricornutum*	10408
Capitella teleta	32070	*Physcomitrella patens*	73923
Capsaspora owczarzaki	8381	*Phytophthora infestans*	17797
Chlamydomonas reinhardtii	31871	*Phytophthora parasitica*	27942
Chlorella variabilis	19733	*Plasmodium falciparum*	5337
Chondrus crispus	9807	*Polysphondylium pallidum*	12367
Chrysemys picta	18838	*Pyropia yezoensis*	1210
Crassostrea gigas	27900	*Reticulomyxa filosa*	39963
Cryptococcus neoformans	7826	*Rhizophagus irregularis*	30312
Cyanidioschyzon merolae	4803	*Rhizopus delemar*	17574
Dictyostelium purpureum	12399	*Saccoglossus kowalevskii*	22093
Echinococcus granulosus	23963	*Salpingoeca rosetta*	11731
Ectocarpus siliculosus	16417	*Saprolegnia diclina*	18229
Edhazardia aedis	4211	*Schistosoma japonicum*	25276
Eimeria tenella	8599	*Selaginella moellendorffii*	69887
Emiliania huxleyi	38555	*Sphaeroforma arctica*	18730
Encephalitozoon cuniculi	6676	*Spironucleus salmonicida*	8333
Entamoeba nuttalli	6187	*Strigomonas culicis*	12083
Enterocytozoon bieneusi	7297	*Tetrahymena thermophila*	24770
Eutrema salsugineum	27741	*Thalassiosira oceanica*	34642
Fonticula alba	6309	*Thecamonas trahens*	10659
Galdieria sulphuraria	7174	*Theileria parva*	4079
Giardia intestinalis	6098	*Toxoplasma gondii*	7987
Guillardia theta	24822	*Trichoplax adhaerens*	11520
Helobdella robusta	23468	*Trypanosoma congolense*	5984
Hydra vulgaris	17795	*Trypanosoma cruzi*	10847
Leishmania major	8316	*Volvox carteri*	14436
Leishmania mexicana	8147	*Zea mays*	62721

Total : 1577333
