# Supplementary material for: IsoSel: Protein Isoform Selector for phylogenetic reconstructions
Source: PLoS One. 2017 Mar 21;12(3):e0174250. doi: 10.1371/journal.pone.0174250 (PMC5360266; doi:10.1371/journal.pone.0174250)
Supplement: S3 Table — For each of the randomly selected human protein, the number of homologous sequences detected using BLASTP is indicated in the third column. The fourth and fifth columns give the gene number and percentage of alternative isoforms, respectively. Alignment length and different statistics about the detected homologs are listed in the last columns. ORFans, datasets containing less than 20 homologs or with only one gene generating alternative isoforms are highlighted in light grey, blue and yellow, respectively. (PDF) [file pone.0174250.s003.pdf]

UniProtKB ID	Gene Name	Number of detected homologs	Number of genes	Percentage of alternative isoforms	Alignment length	Sequences lengths		
						Mean	Median	Maximum
Q96KH6	CR012_HUMAN	1	1	Orphan Genes				
Q3LI68	KR222_HUMAN	1	1					
Q9Y6C7	L3R2A_HUMAN	1	1					
Q9NRJ1	MOST1_HUMAN	1	1					
Q8N339	MT1M_HUMAN	1	1					
Q8TAD7	OCC1_HUMAN	1	1					
Q8IUB9	KR191_HUMAN	1	1					
Q8N1D0	BWR1B_HUMAN	2	1					
Q6ZV80	CB091_HUMAN	2	1					
Q52M58	CN177_HUMAN	2	1					
Q9YNA8	GAK19_HUMAN	2	1					
Q8N446	ZN843_HUMAN	2	1					
Q8NEA5	CS018_HUMAN	4	4	0.00	221	207.25	213	219
Q96NZ9	PRAP1_HUMAN	5	3	60.00	326	207.00	151	319
P06315	KV501_HUMAN	6	5	33.33	121	116.83	117	118
P13500	CCL2_HUMAN	6	6	0.00	133	105.67	100	133
Q7Z7J9	CK2N1_HUMAN	6	6	0.00	79	78.17	78	79
Q9UQF0	SYCY1_HUMAN	6	6	0.00	815	602.5	617.5	686
P03923	NU6M_HUMAN	7	7	0.00	178	174.43	175	177
A6NKF7	TM88B_HUMAN	7	7	0.00	179	170.43	173	178
A8MTZ7	CL071_HUMAN	8	6	50.00	359	312.00	308	347
Q13113	PDZ1I_HUMAN	8	7	25.00	272	142.13	114	272
Q9BT56	SPXN_HUMAN	8	7	25.00	119	113.63	116	119
H3BQB6	STMD1_HUMAN	8	7	25.00	285	264.63	274	279
P26842	CD27_HUMAN	9	8	22.22	276	249.89	257	268
P04141	CSF2_HUMAN	9	8	22.22	155	145.00	144	152
P22466	GALA_HUMAN	9	9	0.00	127	118.56	121	125
Q9UKR8	TSN16_HUMAN	10	6	60.00	308	229.3	241.5	260
Q8NEV9	IL27A_HUMAN	10	9	20.00	252	221.1	232.5	244
Q4G0N7	F229B_HUMAN	10	10	0.00	80	80.00	80	80
Q5JVX7	CA141_HUMAN	13	7	61.54	477	309.31	345	446
P06734	FCER2_HUMAN	13	10	38.46	418	299.31	309	342
Q8NF67	A2012_HUMAN	13	10	46.15	1772	1118.69	992	1726
O00483	NDUA4_HUMAN	13	13	0.00	82	81.92	82	82
O15432	COPT2_HUMAN	14	12	28.57	171	144.21	143	165
Q8IXB3	TUSC5_HUMAN	15	14	13.33	308	183.00	177	297
Q5T9G4	ARM12_HUMAN	18	16	16.67	391	311.00	339	367
O00220	TR10A_HUMAN	19	16	26.32	520	330.05	354	468
Q8IXH6	T53I2_HUMAN	21	14	57.14	326	202.67	219	267
Q5VYV7	SLX4I_HUMAN	21	19	19.05	499	377.52	401	455
Q9NY74	ETAA1_HUMAN	22	18	36.36	1114	864.18	872.5	944
Q8TCU4	ALMS1_HUMAN	24	19	33.33	5613	2360.08	2922	4168
Q9Y547	IFT25_HUMAN	24	21	16.67	322	147.25	140	321
P02750	A2GL_HUMAN	25	21	24.00	488	354.2	342	469
O95073	FSBP_HUMAN	25	23	16.00	2160	362.92	299	2005
Q86XQ3	CTSR3_HUMAN	26	22	30.77	498	377.62	395	454
Q8N6K7	SAMD3_HUMAN	27	18	44.44	580	411.96	510	545
A4D2B0	MBLC1_HUMAN	28	24	28.57	321	227.86	230.5	272
Q9BZ19	ANR60_HUMAN	28	26	14.29	640	301.39	296.5	494
Q9BV99	LRC61_HUMAN	28	26	14.29	580	270.96	259	519
Q9UL15	BAG5_HUMAN	29	25	20.69	569	451.14	450	528
A8MT70	ZBBX_HUMAN	31	18	61.29	988	659.71	733	888
P0C7U1	ASA2B_HUMAN	31	23	38.71	846	673.81	747	785
O00193	SMAP_HUMAN	34	27	32.35	206	166.56	175	232
Q9Y5Z4	HEBP2_HUMAN	35	31	20.00	277	185.43	191	221
P0C7Q5	S35G4_HUMAN	37	36	5.41	441	381.68	411	419
Q9NS73	MBIP1_HUMAN	41	33	24.39	542	315.71	341	381
O14545	TRAD1_HUMAN	44	28	54.55	892	434.25	551	724
Q9NWM3	CUED1_HUMAN	44	31	56.82	543	355.55	378	425
Q2M238	RN3P1_HUMAN	44	35	34.09	881	611.48	617	683
A4D1B5	GSAP_HUMAN	46	31	50.00	1005	607.11	760	891
Q10981	FUT2_HUMAN	47	44	12.77	569	340.87	349	481
O15060	ZBT39_HUMAN	47	45	8.51	1296	702.15	691	1054
Q8IVI9	NOSTN_HUMAN	49	35	42.86	1042	536.2	506	1330
O75414	NDK6_HUMAN	50	38	36.00	239	169.8	177	203
Q8IUI8	CRLF3_HUMAN	50	41	32.00	601	404.42	440	488
Q9NUD5	ZCHC3_HUMAN	50	45	20.00	771	287.44	284	558
P35659	DEK_HUMAN	51	32	60.78	648	344.88	373	548
P52739	ZN131_HUMAN	51	39	35.29	1092	508.57	572	870
Q6IEE8	SN12L_HUMAN	53	38	47.17	1109	714.96	884	946
Q9NVP4	DZAN1_HUMAN	54	39	38.89	1041	635.78	672	803
Q8NA72	POC5_HUMAN	57	40	42.11	1077	534.26	558	721
Q96DZ1	ERLEC_HUMAN	62	49	33.87	702	449.44	479.5	535
Q9H706	GARE1_HUMAN	62	49	38.71	1363	793.77	810	949
Q15526	SURF1_HUMAN	62	53	27.42	942	303.94	291.5	733
Q9H3K2	GHITM_HUMAN	63	54	26.98	555	319.54	345	481
Q6UX53	MET7B_HUMAN	66	59	19.7	295	237.83	244	276
P08567	PLEK_HUMAN	68	61	20.59	770	350.37	352	702
Q7L591	DOK3_HUMAN	70	54	35.71	997	428.69	440	757

UniProtKB ID	Gene Name	Number of detected homologs	Number of genes	Percentage of alternative isoforms	Alignment length	Sequences lengths		
						Mean	Median	Maximum
Q5R3I4	TTC38_HUMAN	70	59	24.29	721	415.99	465.5	615
Q6IED9	DG2L7_HUMAN	71	67	9.86	379	317.25	334	368
A6NDE4	RBY1B_HUMAN	72	52	43.06	1205	383.51	388	1067
Q9BV38	WDR18_HUMAN	73	63	19.18	2735	441.3	431	2469
Q99496	RING2_HUMAN	76	69	14.47	1187	345.49	340.5	935
Q86UD3	MARH3_HUMAN	77	67	19.48	348	226.44	246	287
Q8IWX8	CHERP_HUMAN	78	50	52.56	2380	759.31	884.5	1367
Q9UL16	CFA45_HUMAN	84	69	26.19	2582	507.93	507	1339
Q8WXI4	ACO11_HUMAN	88	63	45.45	959	486.53	555	666
Q8TCX5	RHPN1_HUMAN	89	78	22.47	1983	682.51	675	1182
Q96MB7	HARB1_HUMAN	90	82	15.56	531	303.12	303	432
Q9BX69	CARD6_HUMAN	96	85	20.83	2534	1087.88	926.5	2264
Q14703	MBTP1_HUMAN	96	87	15.63	3130	917.74	1011	1498
Q8N357	S35F6_HUMAN	98	94	8.16	1989	393.79	383	1482
Q8NE31	FA13C_HUMAN	101	71	44.55	1513	761.53	828	1076
Q96GX9	MTNB_HUMAN	103	95	12.62	1610	311.47	242	1073
O00219	HYAS3_HUMAN	108	92	23.15	1153	535.75	552	927
Q8N138	ORML3_HUMAN	108	97	16.67	730	157.49	153	637
Q8WWF6	DNJB3_HUMAN	110	79	40.00	602	262.99	252.5	386
B0YJ81	HACD1_HUMAN	110	96	20.91	516	224.69	227.5	421
Q9BT78	CSN4_HUMAN	113	101	16.81	745	382.65	405	457
P49757	NUMB_HUMAN	114	67	57.02	1964	514.04	591	1325
Q6UXU4	GSG1L_HUMAN	115	84	42.61	603	291.62	297	602
P30408	T4S1_HUMAN	115	96	28.7	295	196.43	201	235
Q9BTU6	P4K2A_HUMAN	115	99	24.35	1899	470.13	466	1207
Q6UWP7	LCLT1_HUMAN	118	100	22.88	1404	364.51	378	716
Q9H0I9	TKTL2_HUMAN	122	102	28.69	899	565.02	624	695
Q9BUB5	MKNK1_HUMAN	123	84	46.34	1908	414.5	425	1142
Q9UJ68	MSRA_HUMAN	126	114	15.08	2034	239.52	209	1420
Q9BV79	MECR_HUMAN	126	115	15.08	1462	364.16	357.5	985
P83916	CBX1_HUMAN	128	108	25.00	436	186.13	185	343
Q8ND56	LS14A_HUMAN	129	79	58.14	978	386.74	422	650
Q8NB12	SMYD1_HUMAN	132	96	43.94	744	429.89	436	502
Q9NRZ7	PLCC_HUMAN	134	114	24.63	2291	363.28	376	1733
B7ZW38	HNRC3_HUMAN	135	86	50.37	556	269.74	290	335
Q9NYZ1	TV23B_HUMAN	135	102	34.07	589	202.21	204	390
Q6N043	Z280D_HUMAN	136	89	49.26	2852	849.77	779	2040
Q92504	S39A7_HUMAN	136	109	30.88	2348	398.73	401.5	969
A6NJ16	IV4F8_HUMAN	138	128	12.32	787	144.2	126	604
Q96NX9	DACH2_HUMAN	143	75	65.73	1542	520.29	548	1081
Q6ZPD9	D19L3_HUMAN	148	111	40.54	1347	659.27	710	801
P54725	RD23A_HUMAN	148	122	27.7	953	365.42	370.5	632
P20618	PSB1_HUMAN	153	151	2.61	580	235.64	237	359
O75376	NCOR1_HUMAN	155	79	60.00	6258	1753.12	2320	3607
Q5GH76	XKR4_HUMAN	155	127	27.74	2568	480.55	447	1547
Q92878	RAD50_HUMAN	155	136	18.71	3451	1028.25	1298	2236
P37802	TAGL2_HUMAN	155	138	20.65	1084	231.1	201	822
Q29RF7	PDS5A_HUMAN	159	122	35.85	5359	1235.22	1357	2228
P34949	MPI_HUMAN	159	133	22.01	1112	390.91	422	567
P00491	PNPH_HUMAN	164	141	24.39	2177	299.27	291	1004
Q8NC42	RN149_HUMAN	165	129	38.18	1840	375.35	392	1371
P52849	NDST2_HUMAN	168	139	27.98	2149	755.57	873	1648
Q5TAX3	TUT4_HUMAN	171	129	35.67	3805	907.11	869	1706
Q7Z429	LFG1_HUMAN	181	148	29.28	2400	302.81	309	1843
O75390	CISY_HUMAN	182	158	17.03	1000	417.43	466	676
P29275	AA2BR_HUMAN	196	168	22.45	1210	340.22	331.5	774
Q96KR1	ZFR_HUMAN	200	134	51.5	2584	755.63	765.5	1104
Q15417	CNN3_HUMAN	203	168	28.08	756	256.16	239	567
P29692	EF1D_HUMAN	210	148	40.48	2691	307.7	237.5	1188
P12955	PEPD_HUMAN	211	193	13.74	1373	459.35	491	707
O75317	UBP12_HUMAN	217	194	19.35	3728	425.55	370	1411
Q9H0J9	PAR12_HUMAN	219	165	39.27	4954	534.79	483	2016
P32322	P5CR1_HUMAN	219	178	26.48	990	286.63	286	551
Q5VST6	AB17B_HUMAN	225	203	17.33	2735	313.65	295	1375
O15127	SCAM2_HUMAN	228	170	42.54	656	287.69	318.5	408
Q96ST3	SIN3A_HUMAN	230	172	35.22	8124	1185.94	1215.5	3776
Q96FT7	ASIC4_HUMAN	232	168	39.66	3045	482.72	515.5	1239
P04440	DPB1_HUMAN	236	129	64.83	939	235.91	253	843
Q8IVL1	NAV2_HUMAN	241	120	68.46	6154	1715.36	1877	2707
Q9Y2D2	S35A3_HUMAN	252	213	25.00	2326	346.06	339.5	1218
Q6Q0C1	S2547_HUMAN	259	224	23.55	1479	305.88	301	628
Q9UPU3	SORC3_HUMAN	274	219	36.13	5950	1065.37	951	2718
Q9UPR5	NAC2_HUMAN	311	197	49.84	3084	801.27	909	1361
Q96S86	HPLN3_HUMAN	335	223	47.16	9692	1035.66	827	3693
Q5JQF8	PAP1M_HUMAN	344	280	28.49	2764	578.16	624	1688
P36268	GGT2_HUMAN	345	276	30.72	2407	524.48	569	902
Q13367	AP3B2_HUMAN	354	287	30.23	3561	899.08	943	1245
Q08AH1	ACSM1_HUMAN	359	305	24.23	2618	606.26	633	1164
Q9Y2H2	SAC2_HUMAN	371	315	22.91	6661	908.5	882	1612

UniProtKB ID	Gene Name	Number of detected homologs	Number of genes	Percentage of alternative isoforms	Alignment length	Sequences lengths		
						Mean	Median	Maximum
P16520	GBB3_HUMAN	398	350	18.59	7073	467.24	344	2929
Q9HCJ2	LRC4C_HUMAN	400	365	15.75	4673	642.41	624	1869
P20023	CR2_HUMAN	444	326	39.41	19676	1771.25	1221.5	5142
Q9HD20	AT131_HUMAN	481	407	22.87	8824	1139.46	1172	1982
P24928	RPB1_HUMAN	485	442	13.61	13324	1473.00	1591	3132
Q03052	PO3F1_HUMAN	486	349	40.53	2249	420.17	381	961
O95258	UCP5_HUMAN	497	440	19.32	1807	305.13	307	1082
Q6PIL6	KCIP4_HUMAN	498	402	28.31	1545	211.44	194	826
P16190	1A33_HUMAN	524	316	55.53	1404	315.81	340	771
P23760	PAX3_HUMAN	553	347	55.15	3389	400.02	403	1328
Q14952	KI2S3_HUMAN	648	325	75.46	1293	416.27	410	841
O00338	ST1C2_HUMAN	651	556	24.73	3925	291.7	293	1341
P34998	CRFR1_HUMAN	655	446	46.11	2563	448.93	444	811
P50995	ANX11_HUMAN	691	554	33.14	5451	382.21	328	1487
Q8IW75	SPA12_HUMAN	729	614	25.65	3423	408.91	407	1983
Q13107	UBP4_HUMAN	737	576	33.38	11046	941.66	963	2519
P05121	PAI1_HUMAN	752	630	26.2	3709	405.95	401	1983
O00478	BT3A3_HUMAN	987	767	35.46	6326	432.82	469	1475
Q16352	AINX_HUMAN	1036	791	36.29	6079	502.00	468	1818
Q9UJA3	MCM8_HUMAN	1040	968	12.12	14942	790.00	799	3503
Q99877	H2B1N_HUMAN	1072	1043	5.13	1220	131.53	126	411
O00212	RHOD_HUMAN	1082	1004	13.03	4962	209.45	194.5	1553
Q9P2N4	ATS9_HUMAN	1101	807	40.6	15320	1115.28	1042	2898
Q02846	GUC2D_HUMAN	1117	963	22.11	16930	804.86	743	2796
Q9NR20	DYRK4_HUMAN	1308	1071	28.44	57681	731.18	597	55569
Q8NGZ2	O14K1_HUMAN	1311	1285	3.81	2692	314.91	313	1287
P31641	SC6A6_HUMAN	1360	930	43.68	8352	590.32	594	1975
Q8N143	BCL6B_HUMAN	1363	1173	22.6	25748	591.83	572	2081
Q00975	CAC1B_HUMAN	1424	795	54.99	21853	1878.55	1978	4695
Q8NGT9	OR2A1_HUMAN	1513	1484	3.64	3098	314.86	314	1287
O00743	PPP6_HUMAN	1518	1352	17.52	9176	374.98	320	1798
P50993	AT1A2_HUMAN	1521	1254	26.36	12723	1010.15	1030	2682
Q8NH81	O10G6_HUMAN	1533	1509	3.13	2855	314.04	313	1287
Q6ZN06	ZN813_HUMAN	1852	1700	13.88	48475	653.73	633	3220
P21506	ZNF10_HUMAN	1856	1712	13.25	45690	619.56	596.5	3220
P58317	ZN121_HUMAN	1860	1704	14.03	43888	611.54	578	3220
Q08881	ITK_HUMAN	1886	1494	31.6	23416	739.05	620.5	5054
P09769	FGR_HUMAN	1888	1507	31.3	23919	724.55	585	5054
Q8N8J6	ZN615_HUMAN	1901	1770	11.31	51347	681.41	661	3220
Q16816	PHKG1_HUMAN	1922	1490	31.95	11984	556.85	516	2268
Q7L7X3	TAOK1_HUMAN	1933	1472	34.56	18488	771.01	690	2735
Q9Y243	AKT3_HUMAN	1936	1564	29.55	13264	638.36	598	2383
