# Supplementary material for: IsoSel: Protein Isoform Selector for phylogenetic reconstructions
Source: PLoS One. 2017 Mar 21;12(3):e0174250. doi: 10.1371/journal.pone.0174250 (PMC5360266; doi:10.1371/journal.pone.0174250)
Supplement: S4 Table — For each of the 154 used datasets, the tree length obtained with each method is listed. The shortest are highlighted in blue. For three datasets (corresponding to proteins Q6ZN06, P58317 and Q8N8J6), the selection with Guidance failed due to program crash. They are highlighted in light orange. (PDF) [file pone.0174250.s004.pdf]

UniProtKB ID	Gene Name	Number of detected homologs	Tree lengths										Best(s) option(s)
			IsoSel parameters							Longest selection	Random selection	GUIDANCE	
			Default	-short	-gap	-GapShort	-DS	-DS -WOT	-auto				
P02750	A2GL_HUMAN	21	4.597446	4.507456	4.598253	4.506655	4.580538	4.584016	4.597446	**4.496752**	4.537022	4.543426	Longest selection
O95073	FSBP_HUMAN	23	2.42282	2.426277	2.391057	2.424949	2.385506	2.387842	**2.384667**	2.424734	2.424084	2.38746	IsoSel
Q86XQ3	CTSR3_HUMAN	22	4.58982	4.692742	4.588927	4.692742	**4.566222**	4.568736	4.592	4.693587	4.707053	4.59219	IsoSel
A4D2B0	MBLC1_HUMAN	24	4.362883	4.364721	4.361022	4.364721	**4.317832**	4.330959	4.362883	4.349941	4.369433	4.361025	IsoSel
Q9BZ19	ANR60_HUMAN	26	5.452709	5.475276	5.442979	5.473921	5.466036	5.477418	**5.442979**	5.478799	5.443144	5.443144	IsoSel
Q9BV99	LRC61_HUMAN	26	4.56561	4.567833	4.565575	4.574061	4.550979	4.57053	**4.549809**	4.568388	4.568536	4.552653	IsoSel
Q9UL15	BAG5_HUMAN	25	2.256033	2.257277	2.266457	**2.253328**	2.256616	2.256033	2.255958	2.257277	2.264002	2.256033	IsoSel
P0C7U1	ASA2B_HUMAN	23	**2.64201**	2.665436	**2.64201**	2.678708	2.658574	2.655057	2.642388	2.793932	2.795248	2.655263	IsoSel
O00193	SMAP_HUMAN	27	**2.587607**	2.658333	2.599961	2.63968	**2.587607**	**2.587607**	2.599961	2.658333	2.806464	**2.587607**	IsoSel GUIDANCE
Q9Y5Z4	HEBP2_HUMAN	31	4.611393	4.604615	4.61161	4.604952	4.612906	4.611393	4.61161	4.600995	**4.598772**	4.616068	Random Selection
Q9NS73	MBIP1_HUMAN	33	5.101701	5.101621	5.096028	5.101701	**5.051151**	5.072093	5.106151	5.08153	5.108792	5.094535	IsoSel
O14545	TRAD1_HUMAN	28	4.864424	5.317183	4.900751	5.319607	4.832188	**4.824785**	4.883533	5.313343	5.158254	4.953175	IsoSel
Q9NWM3	CUED1_HUMAN	31	3.12281	3.207611	3.179241	3.206815	3.119656	**3.11596**	3.122138	3.26747	3.258721	3.197722	IsoSel
Q2M238	RN3P1_HUMAN	35	4.289812	4.290354	4.308641	4.286416	4.292087	4.286335	**4.28507**	4.288334	4.294256	4.304362	IsoSel
A4D1B5	GSAP_HUMAN	31	4.446768	4.616872	4.420859	4.588483	4.359361	4.643253	4.446768	4.60897	**4.329303**	4.605605	Random Selection
Q10981	FUT2_HUMAN	44	6.029055	6.015661	6.062766	6.015661	6.016266	6.026598	6.023406	6.022225	6.051882	**6.007553**	GUIDANCE
O15060	ZBT39_HUMAN	45	4.988531	4.988531	4.996893	4.991607	4.991228	4.99195	4.996893	4.988531	**4.985215**	4.988706	Random Selection
Q8IVI9	NOSTN_HUMAN	35	6.691093	6.78718	6.663993	6.773964	6.744184	6.698953	**6.656511**	6.780574	6.729661	6.66239	IsoSel
O75414	NDK6_HUMAN	38	5.536307	5.589164	5.511277	5.590061	**5.469625**	5.484739	5.551105	5.589368	5.536524	5.583295	IsoSel
Q8IUI8	CRLF3_HUMAN	41	5.646244	5.60291	5.663408	5.595916	**5.588147**	5.593835	5.646244	5.60291	5.92233	**5.588147**	IsoSel GUIDANCE
Q9NUD5	ZCHC3_HUMAN	45	5.790892	5.862762	5.804117	5.852453	5.804117	5.820958	**5.775989**	5.864855	5.848474	5.816673	IsoSel
P35659	DEK_HUMAN	32	3.87691	4.070166	3.923567	4.03708	**3.857294**	3.857368	3.923715	4.075535	4.041046	3.883781	IsoSel
P52739	ZN131_HUMAN	39	4.715988	4.766912	4.712419	4.741605	4.721062	**4.70313**	4.709151	4.757387	4.710047	4.755184	IsoSel
Q6IEE8	SN12L_HUMAN	38	7.061475	7.164119	7.250875	7.166972	7.099706	7.101978	**7.057593**	7.16993	7.140828	7.068993	IsoSel
Q9NVP4	DZAN1_HUMAN	39	8.012714	8.097523	8.063628	8.15251	8.023842	8.025455	**8.010067**	8.134585	8.055366	8.083649	IsoSel
Q8NA72	POC5_HUMAN	40	8.579256	8.604302	8.580949	8.591366	8.56706	**8.5564**	8.604569	8.626998	8.617549	8.584521	IsoSel
Q96DZ1	ERLEC_HUMAN	49	7.311672	7.348616	7.344155	7.326032	7.299984	**7.281132**	7.322238	7.342323	7.340716	7.299722	IsoSel
Q9H706	GARE1_HUMAN	49	4.501105	4.52706	4.506949	4.522643	4.487419	4.486668	4.490761	4.525439	4.548986	**4.486209**	GUIDANCE
Q15526	SURF1_HUMAN	53	9.51477	9.704232	**9.488024**	9.665436	9.518963	9.523593	9.496333	9.715769	9.51711	9.49835	IsoSel
Q9H3K2	GHITM_HUMAN	54	**6.947541**	7.068293	6.95336	7.064707	6.974431	6.969065	6.951332	7.072354	6.978878	6.954927	IsoSel
Q6UX53	MET7B_HUMAN	59	9.000256	9.184999	9.008507	9.003098	8.996037	8.996037	9.000256	9.184955	9.200612	**8.990767**	GUIDANCE
P08567	PLEK_HUMAN	61	6.224589	6.224739	6.218631	6.224589	6.215703	**6.212524**	6.222128	6.224753	6.226494	6.220172	IsoSel
Q7L591	DOK3_HUMAN	54	9.659824	9.763235	9.651194	9.762095	9.636876	9.65717	**9.580077**	9.752913	9.743087	9.683628	IsoSel
Q5R3I4	TTC38_HUMAN	59	9.972182	10.064488	9.981915	10.062306	9.947752	**9.952735**	9.981915	10.062766	10.102877	9.949516	IsoSel
Q6IED9	DG2L7_HUMAN	67	9.055587	9.098683	9.013896	9.101464	9.013896	9.013896	9.055587	9.098683	9.045258	**9.012447**	GUIDANCE
A6NDE4	RBY1B_HUMAN	52	2.345709	2.47035	2.334764	2.471645	2.334815	2.334764	**2.334764**	2.47035	2.50455	2.418828	IsoSel
Q9BV38	WDR18_HUMAN	63	**11.46165**	11.599762	11.482694	11.56774	11.482563	11.4861	11.471829	11.958585	11.557523	11.484143	IsoSel
Q99496	RING2_HUMAN	69	**5.134145**	5.336172	5.175674	5.321936	5.156592	5.137304	5.14362	5.25167	5.199753	5.15691	IsoSel
Q86UD3	MARH3_HUMAN	67	**5.834533**	5.873697	5.853098	5.85937	5.852013	5.852013	5.851489	5.904509	5.906758	**5.834533**	IsoSel GUIDANCE
Q8IWX8	CHERP_HUMAN	50	5.825779	**5.490993**	5.861504	5.658953	5.91026	5.874431	5.937274	5.747056	5.539898	5.598012	IsoSel
Q9UL16	CFA45_HUMAN	69	11.608748	11.697097	11.711672	11.676875	11.605418	11.594697	11.711672	11.693478	11.746452	**11.585413**	GUIDANCE
Q8WXI4	ACO11_HUMAN	63	8.538336	8.727331	8.501399	8.612584	**8.282223**	8.413714	8.493141	8.730924	8.73197	8.674149	IsoSel
Q8TCX5	RHPN1_HUMAN	78	**11.597525**	11.623274	11.676172	11.625651	11.654015	11.66752	11.679195	11.646576	11.646887	11.660727	IsoSel
Q96MB7	HARB1_HUMAN	82	8.836914	8.925327	8.909699	8.881635	8.830503	8.830973	8.854603	8.883871	8.885663	**8.828312**	GUIDANCE
Q9BX69	CARD6_HUMAN	85	11.87574	11.935126	11.875929	11.954234	**11.704604**	11.798093	11.883497	11.932635	11.947354	11.880483	IsoSel
Q14703	MBTP1_HUMAN	87	12.021779	12.145369	11.953181	12.157947	12.002797	**11.939721**	11.982016	12.185414	12.545194	12.018542	IsoSel
Q8N357	S35F6_HUMAN	94	19.124105	**19.044761**	19.122809	19.100934	19.059396	19.094502	19.087911	19.045363	19.116137	19.066564	IsoSel
Q8NE31	FA13C_HUMAN	71	8.720148	8.968506	8.877483	8.916869	8.715765	**8.707377**	8.867282	8.95805	9.029283	8.713951	IsoSel
Q96GX9	MTNB_HUMAN	95	12.593975	12.65068	**12.56399**	12.66678	12.624359	12.649828	12.600184	12.657305	12.604003	12.602532	IsoSel
O00219	HYAS3_HUMAN	92	8.003847	8.122643	8.076231	8.123316	7.994712	**7.991722**	8.076077	8.125303	8.125215	8.001156	IsoSel
Q8N138	ORML3_HUMAN	97	5.864354	5.958232	5.858626	5.939646	5.929704	5.929704	**5.858626**	5.957428	5.926947	5.864576	IsoSel
Q8WWF6	DNJB3_HUMAN	79	**8.950812**	9.328478	9.001258	9.280247	9.00827	9.010984	8.992874	9.484479	9.193623	8.971014	IsoSel
B0YJ81	HACD1_HUMAN	96	12.549739	12.564792	12.505846	12.549979	**12.402477**	**12.402477**	12.554831	12.53625	12.678993	12.494177	IsoSel
Q9BT78	CSN4_HUMAN	101	13.727894	13.785228	13.733542	13.74443	**13.719834**	13.721384	13.735676	13.784009	13.726503	13.72513	IsoSel
P49757	NUMB_HUMAN	67	7.499549	7.908585	7.54671	7.957006	7.439821	**7.413247**	7.539584	7.931879	7.691481	7.444785	IsoSel
Q6UXU4	GSG1L_HUMAN	84	8.381121	8.351501	8.275085	8.352118	8.158555	**8.133426**	8.275147	8.351838	8.484351	8.163589	IsoSel
P30408	T4S1_HUMAN	96	12.789275	12.878486	12.84339	12.859244	**12.746798**	12.760169	12.781165	12.911501	12.860242	12.782832	IsoSel
Q9BTU6	P4K2A_HUMAN	99	11.924324	11.931491	**11.871417**	11.946731	11.896324	11.875177	11.88859	11.979953	11.919957	11.884249	IsoSel
Q6UWP7	LCLT1_HUMAN	100	17.127196	17.310811	17.133423	17.287116	17.19319	17.162137	**17.122654**	17.291595	17.292616	17.149201	IsoSel
Q9H0I9	TKTL2_HUMAN	102	10.049058	10.11714	10.121561	10.101428	10.041571	**10.033317**	10.046812	10.116074	10.063763	10.088059	IsoSel
Q9BUB5	MKNK1_HUMAN	84	10.608813	10.726413	10.641146	10.616099	10.598808	**10.593859**	10.628666	10.744767	11.040727	10.596668	IsoSel
Q9UJ68	MSRA_HUMAN	114	14.685568	14.872788	**14.584424**	14.830693	14.682188	14.680843	14.620681	14.861709	14.809256	14.69758	IsoSel
Q9BV79	MECR_HUMAN	115	21.076278	21.161949	**21.015759**	21.119689	21.062796	21.072839	21.041765	21.159108	21.118822	21.076539	IsoSel
P83916	CBX1_HUMAN	108	8.823219	8.884958	8.875151	**8.783338**	8.800616	8.824856	8.87746	8.935631	8.801719	8.811087	IsoSel
Q8ND56	LS14A_HUMAN	79	8.857895	9.325648	8.922942	9.353603	8.941352	8.905345	8.911229	9.38491	9.493119	**8.829995**	GUIDANCE
Q8NB12	SMYD1_HUMAN	96	12.653515	12.699595	12.828112	12.661377	12.679084	12.731902	12.653515	12.752742	12.849626	**12.634977**	GUIDANCE
Q9NRZ7	PLCC_HUMAN	114	**14.448895**	14.499527	14.582266	14.501576	14.474756	14.463644	14.58118	14.51498	14.501038	14.55235	IsoSel
B7ZW38	HNRC3_HUMAN	86	6.344407	6.765392	6.297719	6.676006	6.359691	6.375433	**6.295021**	6.748371	6.701264	6.338956	IsoSel
Q9NYZ1	TV23B_HUMAN	102	14.201254	14.175272	14.113014	14.166257	**14.074866**	14.103124	14.10404	14.148029	14.16232	14.240676	IsoSel
Q6N043	Z280D_HUMAN	89	**13.044935**	13.132516	13.081605	13.093362	13.058265	13.078018	13.055444	13.124049	13.120397	13.059674	IsoSel
Q92504	S39A7_HUMAN	109	16.67428	16.840961	16.834769	16.759807	**16.671115**	16.690452	16.821369	16.780755	17.045807	16.679997	IsoSel
A6NJ16	IV4F8_HUMAN	128	11.090596	11.094973	11.102533	11.108106	11.099391	**11.078177**	11.097823	11.088835	11.13335	11.080364	IsoSel
Q96NX9	DACH2_HUMAN	75	6.843571	6.783356	6.963176	7.008006	6.755688	**6.494218**	6.965435	6.724478	6.941486	6.671817	IsoSel
Q6ZPD9	D19L3_HUMAN	111	15.318592	15.397942	15.407713	15.408515	15.388817	15.431884	15.395204	15.408634	15.429439	**15.307738**	GUIDANCE
P54725	RD23A_HUMAN	122	16.468806	16.356608	16.355015	16.3653	**16.257276**	16.29149	16.332837	16.410976	16.442663	16.289379	IsoSel
P20618	PSB1_HUMAN	151	19.505141	19.517114	19.483838	19.515072	19.515054	19.502933	19.50061	**19.422864**	19.526563	19.524562	Longest selection
O75376	NCOR1_HUMAN	79	10.509141	10.332135	10.603259	10.713218	10.448386	**10.134603**	10.524293	10.687477	10.338303	10.47888	IsoSel
Q5GH76	XKR4_HUMAN	127	**13.72337**	14.011101	13.7246	13.854964	13.750461	13.738786	13.731109	14.044447	13.779697	13.908635	IsoSel
Q92878	RAD50_HUMAN	136	27.262826	26.99967	27.260264	27.046351	27.011617	27.172555	27.245646	**26.985054**	27.067777	27.251454	Longest selection
P37802	TAGL2_HUMAN	138	11.846311	11.792424	11.881464	11.809042	11.736138	**11.728037**	11.877667	11.785545	11.767206	11.76416	IsoSel

UniProtKB ID	Gene Name	Number of detected homologs	Tree lengths										Best(s) option(s)
			IsoSel parameters							Longest selection	Random selection	GUIDANCE	
			Default	-short	-gap	-GapShort	-DS	-DS -WOT	-auto				
Q29RF7	PDS5A_HUMAN	122	16.681303	16.805721	16.666657	16.797869	16.640132	**16.603304**	16.663495	16.782967	16.72884	16.604533	IsoSel
P34949	MPI_HUMAN	133	**24.338607**	24.425408	24.35664	24.42967	24.390106	24.380715	24.38063	24.429772	24.413128	24.346626	IsoSel
P00491	PNPH_HUMAN	141	20.660833	20.666886	20.640589	**20.606039**	20.618159	20.698338	20.641285	20.654653	20.62934	20.615428	IsoSel
Q8NC42	RN149_HUMAN	129	14.636192	14.626097	14.654939	14.699919	**14.540734**	14.568015	14.627182	14.621603	14.644456	14.648219	IsoSel
P52849	NDST2_HUMAN	139	**10.836273**	10.907749	11.14472	10.913726	10.876582	10.875802	11.163408	10.893912	11.080566	10.867553	IsoSel
Q5TAX3	TUT4_HUMAN	129	**20.280145**	20.673762	20.39692	20.664762	20.305878	20.30333	20.432609	20.6321	20.554853	20.488628	IsoSel
Q7Z429	LFG1_HUMAN	148	21.38022	21.794027	21.385631	21.655543	21.50708	21.447418	21.356382	21.660441	21.642069	**21.30877**	GUIDANCE
O75390	CISY_HUMAN	158	16.800899	16.759669	16.759256	16.768189	**16.707539**	16.720023	16.769855	16.781765	16.776782	16.725767	IsoSel
P29275	AA2BR_HUMAN	168	24.543886	24.632925	24.64411	24.688255	24.43424	**24.431672**	24.598118	24.652063	24.696328	24.551636	IsoSel
Q96KR1	ZFR_HUMAN	134	**14.036474**	14.480758	14.050356	14.339119	14.337305	14.250977	14.055419	14.376137	14.427427	14.113895	IsoSel
Q15417	CNN3_HUMAN	168	14.574308	14.715999	14.659278	14.723673	14.71931	14.572738	14.659106	14.733766	14.751294	**14.557473**	GUIDANCE
P29692	EF1D_HUMAN	148	15.786259	16.449171	15.760553	16.506604	15.801723	15.737246	15.743147	16.593961	16.393981	**15.694989**	GUIDANCE
P12955	PEPD_HUMAN	193	31.125584	30.963373	31.149821	30.991768	30.872505	**30.85341**	31.15904	30.986585	30.952952	31.080363	IsoSel
O75317	UBP12_HUMAN	194	**19.396765**	19.491878	19.413562	19.517208	19.413067	19.505667	19.405418	19.510985	19.48158	19.436603	IsoSel
Q9H0J9	PAR12_HUMAN	165	28.551336	29.02181	28.477892	28.861047	28.574376	28.535246	28.477892	29.043642	28.737708	**28.324942**	GUIDANCE
P32322	P5CR1_HUMAN	178	**25.483622**	25.697041	25.650561	25.709951	25.544261	25.543917	25.609667	25.762489	25.605219	25.665335	IsoSel
Q5VST6	AB17B_HUMAN	203	**20.146173**	20.310403	20.165165	20.217121	20.198414	20.219096	20.16021	20.24217	20.345521	20.258275	IsoSel
O15127	SCAM2_HUMAN	170	**17.732535**	17.966834	17.901149	17.971685	17.804589	17.811008	17.910171	18.02793	17.982901	17.810263	IsoSel
Q96ST3	SIN3A_HUMAN	172	24.967064	25.002895	24.885669	25.065141	24.854713	**24.76163**	24.894717	24.989958	24.92811	24.914454	IsoSel
Q96FT7	ASIC4_HUMAN	168	21.484736	21.977053	21.560532	21.841942	**21.249601**	21.327165	21.55916	22.053073	21.942774	21.496679	IsoSel
P04440	DPB1_HUMAN	129	12.987724	13.086557	13.049182	13.000796	12.958908	13.013863	13.050024	13.095758	13.23249	**12.880371**	GUIDANCE
Q8IVL1	NAV2_HUMAN	120	12.429949	12.374398	12.148624	12.72133	12.648649	12.017341	12.149669	12.447574	12.759682	**11.898085**	GUIDANCE
Q9Y2D2	S35A3_HUMAN	213	32.63223	33.009292	32.696471	32.883196	**32.566101**	32.583487	32.689373	33.099069	32.95183	32.746737	IsoSel
Q6Q0C1	S2547_HUMAN	224	31.875783	32.091473	31.909091	31.907539	**31.774348**	31.777598	31.926374	32.172846	31.984385	31.806433	IsoSel
Q9UPU3	SORC3_HUMAN	219	30.663297	30.574281	30.470612	30.480505	30.421675	**30.327493**	30.465047	30.824749	30.642418	30.619921	IsoSel
Q9UPR5	NAC2_HUMAN	197	23.259195	23.946571	23.314432	23.628691	23.316935	23.321093	23.269444	23.936093	23.774362	**23.183944**	GUIDANCE
Q96S86	HPLN3_HUMAN	223	29.454727	31.772291	29.440932	30.897622	29.38021	**29.257858**	29.389011	31.883287	30.563114	29.551961	IsoSel
Q5JQF8	PAP1M_HUMAN	280	26.680405	27.336541	26.770204	27.127948	26.724819	26.718565	26.76526	27.121283	27.062228	**26.655286**	GUIDANCE
P36268	GGT2_HUMAN	276	48.635521	48.947747	48.696766	48.867379	48.817907	48.799968	48.707533	48.973588	48.93513	**48.626352**	GUIDANCE
Q13367	AP3B2_HUMAN	287	35.083737	35.584704	35.296066	35.662221	**34.948032**	35.188522	35.183238	35.695017	35.291859	35.419859	IsoSel
Q08AH1	ACSM1_HUMAN	305	**39.577208**	39.83002	39.668478	39.817197	39.592017	39.61631	39.663693	39.905387	39.808993	39.584787	IsoSel
Q9Y2H2	SAC2_HUMAN	315	**52.842341**	53.291535	53.136048	53.445172	52.936519	53.148551	53.077146	53.271751	52.909184	53.077231	IsoSel
P16520	GBB3_HUMAN	350	36.103932	36.323154	36.174257	36.222643	36.220434	36.167137	36.175003	36.340637	36.362035	**36.0277**	GUIDANCE
Q9HCJ2	LRC4C_HUMAN	365	42.066584	42.120381	**41.957855**	42.145077	42.124164	42.114189	41.960869	42.204054	42.014564	42.02776	IsoSel
P20023	CR2_HUMAN	326	61.09517	61.240011	61.249571	61.340523	61.332624	61.369748	61.29702	61.179051	61.302828	**61.0642**	GUIDANCE
Q9HD20	AT131_HUMAN	407	67.617449	67.488846	67.580906	67.700848	67.594888	67.581894	67.595092	67.535062	**67.436447**	67.539763	Random Selection
P24928	RPB1_HUMAN	442	**61.611975**	61.848775	61.94107	61.783527	61.843286	61.849403	61.838337	61.888112	61.689076	61.940836	IsoSel
Q03052	PO3F1_HUMAN	349	25.233457	25.978432	25.255889	25.791298	**25.219729**	25.389986	25.272388	26.112429	25.587636	25.36597	IsoSel
O95258	UCP5_HUMAN	440	55.778903	55.836512	55.963691	**55.676476**	55.85779	55.869266	55.940406	55.928908	55.83401	55.765726	IsoSel
Q6PIL6	KCIP4_HUMAN	402	26.998903	27.00019	26.949076	27.013321	26.837852	26.756309	26.974522	27.218135	27.230944	26.863963	IsoSel
P16190	1A33_HUMAN	316	39.722929	39.99909	39.838939	40.037313	**39.448533**	39.60992	39.845915	39.825643	40.119478	39.741112	IsoSel
P23760	PAX3_HUMAN	347	27.121197	28.297297	27.037682	28.084991	26.837411	26.637067	26.999074	28.272222	27.58785	**26.55202**	GUIDANCE
Q14952	KI2S3_HUMAN	325	**7.617723**	7.793681	7.794053	7.778462	7.75449	7.693322	7.756214	7.876233	7.838423	7.726719	IsoSel
O00338	ST1C2_HUMAN	556	83.176793	83.276218	83.175141	83.22037	83.070691	83.060698	83.070691	83.357911	83.50571	**83.007004**	GUIDANCE
P34998	CRFR1_HUMAN	446	51.385883	52.702645	51.670818	52.595048	51.446718	51.422331	51.413626	52.970605	51.935478	**51.37507**	GUIDANCE
P50995	ANX11_HUMAN	691	69.497071	69.55111	69.835302	69.562837	**69.170679**	69.191873	**69.170679**	69.646872	69.955055	69.318044	IsoSel
Q8IW75	SPA12_HUMAN	614	96.191074	96.321382	96.31813	96.341132	96.116036	96.126064	**96.116036**	96.396375	96.460724	96.138631	IsoSel
Q13107	UBP4_HUMAN	576	91.639254	93.130601	91.994432	92.798137	92.033625	91.969388	92.033625	93.168562	92.945573	**91.509436**	GUIDANCE
P05121	PAI1_HUMAN	630	95.42923	95.633289	95.530808	95.550167	95.469099	**95.386896**	95.469099	95.550706	95.603546	95.461598	IsoSel
O00478	BT3A3_HUMAN	767	**120.684603**	121.251474	121.079384	121.170267	120.956867	120.92001	120.956867	121.590853	121.571826	120.736794	IsoSel
Q16352	AINX_HUMAN	791	86.737551	87.519177	86.881317	87.024765	86.709446	86.631382	86.709446	87.543183	87.346208	**86.458886**	GUIDANCE
Q9UJA3	MCM8_HUMAN	968	139.287172	139.628694	**139.283269**	139.438454	139.341627	139.330169	139.341627	139.509259	139.394613	139.384708	IsoSel
Q99877	H2B1N_HUMAN	1043	20.841571	**20.738172**	20.861827	20.824657	20.777539	20.777539	20.777539	20.932359	20.934263	20.948642	IsoSel
O00212	RHOD_HUMAN	1004	79.353151	79.466918	79.53243	79.57797	79.005872	**79.005804**	79.005872	79.651105	79.512511	79.016914	DSWOT
Q9P2N4	ATS9_HUMAN	807	102.442404	104.494226	103.375973	104.829332	103.259666	103.339791	103.259666	104.802206	103.89052	**102.226567**	GUIDANCE
Q02846	GUC2D_HUMAN	963	144.932522	146.07098	144.759312	146.226925	144.194761	144.194761	**144.194761**	146.543635	145.928729	144.666766	IsoSel
Q9NR20	DYRK4_HUMAN	1071	139.030111	140.063459	138.744996	140.052342	138.724908	**138.693342**	138.724908	140.200571	139.853354	138.934719	IsoSel
Q8NGZ2	O14K1_HUMAN	1285	**142.63909**	142.660765	**142.63909**	142.684104	142.660391	142.655685	142.660391	142.684112	142.649038	142.677251	IsoSel
P31641	SC6A6_HUMAN	930	117.380102	118.114877	117.028664	118.026841	117.010008	**116.819419**	117.010008	118.492406	118.061746	116.9312	IsoSel
Q8N143	BCL6B_HUMAN	1173	178.415248	179.049332	178.729485	178.832209	177.912509	**177.868797**	177.912509	178.573046	179.025722	178.709398	IsoSel
Q00975	CAC1B_HUMAN	795	101.295019	102.821724	101.270898	101.851208	101.466898	101.661889	101.466898	102.266346	102.315461	**101.023797**	GUIDANCE
Q8NGT9	OR2A1_HUMAN	1484	147.754127	147.746223	147.754127	147.810474	147.725061	147.725061	147.725061	147.753181	**147.713905**	147.763244	Random Selection
O00743	PPP6_HUMAN	1352	102.877702	103.212556	103.15491	103.124734	102.846453	**102.813638**	102.846453	103.516391	103.37032	102.824552	DSWOT
P50993	AT1A2_HUMAN	1254	139.050708	139.342866	139.126277	139.402929	138.858952	139.047814	**138.858952**	139.299023	139.457356	139.324251	IsoSel
Q8NH81	O10G6_HUMAN	1509	147.32696	147.302459	147.32696	147.292922	147.232307	147.232307	**147.232307**	147.256568	147.239875	147.234101	IsoSel
Q6ZN06	ZN813_HUMAN	1700	274.578547	273.993383	**273.75277**	274.386215	274.703226	275.230985	274.703226	274.790417	275.255475		
P21506	ZNF10_HUMAN	1712	263.670597	263.569747	262.994475	263.65716	262.976791	**262.706257**	262.976791	263.762862	263.020695	264.55002	IsoSel
P58317	ZN121_HUMAN	1704	257.861688	258.290748	257.80475	257.709389	258.045684	257.757963	258.045684	**257.599248**	258.09025		
Q08881	ITK_HUMAN	1494	210.250729	210.882114	**210.129451**	210.856231	210.242479	210.195978	210.242479	211.40288	210.888958	210.235524	IsoSel
P09769	FGR_HUMAN	1507	211.187211	211.758859	210.767656	211.665657	210.742845	**210.289655**	210.742845	211.644695	211.787395	210.667359	IsoSel
Q8N8J6	ZN615_HUMAN	1770	291.587772	291.442636	**290.851263**	291.631889	291.688245	291.633022	291.688245	292.340218	292.644415		
Q16816	PHKG1_HUMAN	1490	156.762933	157.978997	157.047068	157.930677	156.811918	156.915618	156.811918	158.393914	157.623469	**156.691418**	GUIDANCE
Q7L7X3	TAOK1_HUMAN	1472	197.207757	199.953857	197.611329	199.828995	198.12465	198.049758	198.12465	200.241339	198.988405	**197.095021**	GUIDANCE
Q9Y243	AKT3_HUMAN	1564	180.221158	181.119378	179.916022	180.922849	179.773283	179.820357	**179.773283**	181.154473	180.881436	180.024243	IsoSel
		**Means :**	**36.90**	**37.15**	**36.94**	**37.12**	**36.88**	**36.87**	**36.91**	**37.19**	**37.11**	**36.89**	
