# Supplementary material for: IsoSel: Protein Isoform Selector for phylogenetic reconstructions
Source: PLoS One. 2017 Mar 21;12(3):e0174250. doi: 10.1371/journal.pone.0174250 (PMC5360266; doi:10.1371/journal.pone.0174250)
Supplement: S5 Table — For each of the 154 used datasets, the DL score computed by Notung with each isoform selection strategy is listed. The most consistent are highlighted in blue. For three datasets (corresponding to proteins Q6ZN06, P58317 and Q8N8J6), the selection with Guidance failed due to program crash. They are highlighted in light orange. (PDF) [file pone.0174250.s005.pdf]

UniProtKB ID	Gene Name	Number of detected homologs	Tree lengths										Best(s) option(s)
			IsoSel parameters							Longest selection	Random selection	GUIDANCE	
			Default	-short	-gap	-GapShort	-DS	-DS -WOT	-auto				
P02750	A2GL_HUMAN	21	35	**30.5**	35	35	35	35	35	**30.5**	35	35	IsoSel Longest selection
O95073	FSBP_HUMAN	23	35	34	**31.5**	34	32.5	**31.5**	**31.5**	34	34	**31.5**	IsoSel GUIDANCE
Q86XQ3	CTSR3_HUMAN	22	42	**37.5**	42	**37.5**	42	42	42	**37.5**	47	42	IsoSel Longest selection
A4D2B0	MBLC1_HUMAN	24	**50.5**	58	52.5	58	58	58	**50.5**	58	58	52.5	IsoSel
Q9BZ19	ANR60_HUMAN	26	**59**	60	60	60	60	60	60	60	60	60	IsoSel
Q9BV99	LRC61_HUMAN	26	43	**35.5**	43	**35.5**	36.5	37.5	36.5	**35.5**	46.5	36.5	IsoSel Longest selection
Q9UL15	BAG5_HUMAN	25	**31.5**	**31.5**	43.5	**31.5**	**31.5**	**31.5**	**31.5**	**31.5**	43.5	**31.5**	IsoSel Longest selection GUIDANCE
P0C7U1	ASA2B_HUMAN	23	33	**27.5**	33	27.5	29.5	29.5	33	**27.5**	28.5	29.5	IsoSel Longest selection
O00193	SMAP_HUMAN	27	45.5	45.5	45.5	45.5	45.5	45.5	45.5	45.5	**39**	45.5	Random selection
Q9Y5Z4	HEBP2_HUMAN	31	**70**	77.5	**70**	77.5	75.5	**70**	**70**	77.5	77.5	**70**	IsoSel GUIDANCE
Q9NS73	MBIP1_HUMAN	33	69.5	69.5	**54**	69.5	68.5	69.5	64	70.5	69.5	68.5	IsoSel
O14545	TRAD1_HUMAN	28	42	**40**	42	**40**	42	42	**40**	**40**	49	46.5	IsoSel Longest selection
Q9NWM3	CUED1_HUMAN	31	**40**	44.5	47.5	54.5	56.5	61	**40**	46.5	53	45.5	IsoSel
Q2M238	RN3P1_HUMAN	35	66	**60.5**	66	**60.5**	66	66	66	**60.5**	**60.5**	63	IsoSel Longest and Random selections
A4D1B5	GSAP_HUMAN	31	38.5	42	48.5	43	41	42	38.5	42	42	**37.5**	GUIDANCE
Q10981	FUT2_HUMAN	44	157	**120.5**	158.5	158.5	124	124	124	**120.5**	**120.5**	124	IsoSel Longest and Random selections
O15060	ZBT39_HUMAN	45	106	106	104	104	104	104	104	101.5	**99.5**	104	Random selection
Q8IVI9	NOSTN_HUMAN	35	38.5	40.5	39.5	40.5	**34**	39.5	39.5	44	38.5	39.5	IsoSel
O75414	NDK6_HUMAN	38	137	**115.5**	138	131.5	138	135	137	**115.5**	133.5	**115.5**	IsoSel Longest selection GUIDANCE
Q8IUI8	CRLF3_HUMAN	41	55	50.5	50.5	50.5	50.5	50.5	55	50.5	**47**	50.5	Random selection
Q9NUD5	ZCHC3_HUMAN	45	91	92	91	91	**84.5**	91	91	91	**84.5**	**84.5**	IsoSel Random selection GUIDANCE
P35659	DEK_HUMAN	32	63.5	64	62	68.5	63.5	63.5	64	64	65	**60.5**	GUIDANCE
P52739	ZN131_HUMAN	39	65.5	75.5	65.5	65	65.5	65.5	70	73	**60**	69	Random selection
Q6IEE8	SN12L_HUMAN	38	96.5	99	99	99	**87**	**87**	96.5	99	93.5	96.5	IsoSel
Q9NVP4	DZAN1_HUMAN	39	**45**	54	57	54	**45**	**45**	**45**	53	49.5	49.5	IsoSel
Q8NA72	POC5_HUMAN	40	61	66.5	56.5	50.5	72	**41.5**	52	61	83.5	**41.5**	IsoSel GUIDANCE
Q96DZ1	ERLEC_HUMAN	49	93	90.5	**84.5**	89.5	86.5	100.5	95.5	95	87.5	95.5	IsoSel
Q9H706	GARE1_HUMAN	49	79.5	**71**	87	76.5	99.5	98	85	72	133	73	IsoSel
Q15526	SURF1_HUMAN	53	171.5	159.5	162.5	159.5	173.5	**151.5**	173	171.5	172.5	171.5	IsoSel
Q9H3K2	GHITM_HUMAN	54	179.5	**103.5**	183.5	107	175	179.5	175	108	166	175	IsoSel
Q6UX53	MET7B_HUMAN	59	131.5	**126.5**	136.5	131.5	136.5	136.5	130.5	**126.5**	128.5	131.5	IsoSel Longest selection
P08567	PLEK_HUMAN	61	**98**	**98**	**98**	108	**98**	**98**	**98**	**98**	**98**	**98**	All
Q7L591	DOK3_HUMAN	54	103.5	102.5	**95.5**	101	101	120.5	106.5	101	119.5	117.5	IsoSel
Q5R3I4	TTC38_HUMAN	59	**141**	146	**141**	146	143	143	143	**141**	**141**	**141**	All
Q6IED9	DG2L7_HUMAN	67	145	145	145	145	145	145	145	**138.5**	145	145	Longest selection
A6NDE4	RBY1B_HUMAN	52	117	122.5	114	116	119	118	111.5	120.5	**108.5**	128	Random selection
Q9BV38	WDR18_HUMAN	63	142	133	142	140	139	136.5	139.5	**131**	138.5	149.5	Longest selection
Q99496	RING2_HUMAN	69	184.5	178	207	183.5	191	187.5	191	**143.5**	173.5	160.5	Longest selection
Q86UD3	MARH3_HUMAN	67	184.5	180	193	180	181.5	181.5	193	177	**163**	184.5	Random selection
Q8IWX8	CHERP_HUMAN	50	85	86	92.5	82.5	**70.5**	94.5	108.5	77.5	95	79.5	IsoSel
Q9UL16	CFA45_HUMAN	69	87	79	84.5	91	79	79	84.5	79	**70**	83.5	Random selection
Q8WXI4	ACO11_HUMAN	63	93.5	101.5	**90**	92.5	96.5	92	90	96	103.5	97	IsoSel
Q8TCX5	RHPN1_HUMAN	78	79.5	88.5	79.5	113.5	**72**	74.5	80.5	122.5	79.5	85.5	IsoSel
Q96MB7	HARB1_HUMAN	82	174	186	175	175	174	174	174	**169.5**	174	179.5	Longest selection
Q9BX69	CARD6_HUMAN	85	146.5	144	146.5	**136.5**	152.5	144	154	**136.5**	140	154	IsoSel Longest selection
Q14703	MBTP1_HUMAN	87	232	197	217	186	229.5	205.5	208.5	**180.5**	206	193	Longest selection
Q8N357	S35F6_HUMAN	94	249.5	247	264.5	**232**	256	260	249.5	236.5	244	239.5	IsoSel
Q8NE31	FA13C_HUMAN	71	100	88.5	100	111.5	98	104	**81**	92	99.5	99	IsoSel
Q96GX9	MTNB_HUMAN	95	272	280.5	281.5	**253**	279.5	282.5	295.5	276.5	280.5	273.5	IsoSel
O00219	HYAS3_HUMAN	92	187.5	188	192.5	187	192	185.5	**180.5**	**180.5**	183.5	200	IsoSel Longest selection
Q8N138	ORML3_HUMAN	97	262.5	228	**222.5**	257	261.5	261.5	243.5	243.5	225	262	IsoSel
Q8WWF6	DNJB3_HUMAN	79	295.5	285	268	273	267.5	263.5	260	**238**	273.5	279.5	Longest selection
B0YJ81	HACD1_HUMAN	96	261.5	273.5	291.5	274.5	299	295	271.5	261.5	**248.5**	**248.5**	Random selection GUIDANCE
Q9BT78	CSN4_HUMAN	101	236.5	222	241	232	228	**207**	221	235.5	231.5	223	IsoSel
P49757	NUMB_HUMAN	67	140	141	135	144.5	145	142.5	**130.5**	134.5	134.5	144.5	IsoSel
Q6UXU4	GSG1L_HUMAN	84	151.5	158.5	173	164	**150.5**	156	173	158.5	163.5	**150.5**	IsoSel GUIDANCE
P30408	T4S1_HUMAN	96	260	276.5	**248.5**	263.5	263.5	263.5	266.5	276.5	265	259	IsoSel
Q9BTU6	P4K2A_HUMAN	99	209.5	202	196	208	**183.5**	199	178	223	210.5	214.5	IsoSel
Q6UWP7	LCLT1_HUMAN	100	219.5	208.5	219.5	208.5	210	210	201.5	**191**	208	212	Longest selection
Q9H0I9	TKTL2_HUMAN	102	192	198.5	195	199.5	187.5	326.5	192	198.5	**186.5**	195	Random selection
Q9BUB5	MKNK1_HUMAN	84	199	178	193.5	173	**169**	171	192.5	171	176.5	182	IsoSel
Q9UJ68	MSRA_HUMAN	114	420	414.5	416.5	425.5	420	435.5	**296**	425.5	425.5	411	IsoSel
Q9BV79	MECR_HUMAN	115	329	322.5	346	314	313	332	350.5	**291.5**	326.5	323.5	Longest selection
P83916	CBX1_HUMAN	108	274.5	264	260	253.5	262.5	274.5	283	**247**	270	293.5	Longest selection
Q8ND56	LS14A_HUMAN	79	170	164	179	**140**	165.5	172.5	166	158	195.5	164.5	IsoSel
Q8NB12	SMYD1_HUMAN	96	243	221	248.5	227.5	223	251	243	**217.5**	237.5	243.5	Longest selection
Q9NRZ7	PLCC_HUMAN	114	213.5	**180**	196	**180**	181	182	189.5	**180**	190.5	191.5	IsoSel Longest selection
B7ZW38	HNRC3_HUMAN	86	193	195	198	202	220	210	216.5	193	**189**	207	Random selection
Q9NYZ1	TV23B_HUMAN	102	222.5	229.5	259	229.5	**209.5**	212	228	221.5	222.5	269	IsoSel
Q6N043	Z280D_HUMAN	89	**139**	180.5	177.5	158.5	153.5	134.5	158.5	140.5	164	142.5	IsoSel
Q92504	S39A7_HUMAN	109	271.5	300	284	304	273.5	**256.5**	270	296	286.5	261.5	IsoSel
A6NJ16	IV4F8_HUMAN	128	260.5	222.5	249	280.5	246.5	**211**	277	274.5	283	213.5	IsoSel
Q96NX9	DACH2_HUMAN	75	224.5	173	188	170	209.5	219	178	**160.5**	166	190	Longest selection
Q6ZPD9	D19L3_HUMAN	111	**184.5**	195.5	188.5	196.5	192	197	187.5	187.5	186	192	IsoSel
P54725	RD23A_HUMAN	122	226.5	269	286.5	240.5	247	259	259.5	285	**210**	236	Random selection
P20618	PSB1_HUMAN	151	319.5	303	309.5	307	290	**273**	307	318	295.5	313.5	IsoSel
O75376	NCOR1_HUMAN	79	149	105	124.5	123.5	165.5	165	132	**96.5**	148.5	140	Longest selection
Q5GH76	XKR4_HUMAN	127	265	258	266	280	275	263	283.5	**256.5**	267.5	272	Longest selection
Q92878	RAD50_HUMAN	136	**254**	293	319	281	290.5	278.5	315	287.5	315.5	260.5	IsoSel
P37802	TAGL2_HUMAN	138	397.5	409.5	409	404	398	**391.5**	405.5	395.5	406.5	397.5	IsoSel
Q29RF7	PDS5A_HUMAN	122	203.5	281	285	**187**	199	247	223.5	282.5	193.5	279	IsoSel
P34949	MPI_HUMAN	133	311.5	**272**	304	305.5	301	308	324	326.5	313	309	IsoSel
P00491	PNPH_HUMAN	141	**325**	335.5	354	350	346.5	343.5	346	330	343.5	363	IsoSel
Q8NC42	RN149_HUMAN	129	278	**257**	257.5	260.5	263	268.5	271	263.5	291.5	302.5	IsoSel
P52849	NDST2_HUMAN	139	299	301.5	322	273.5	298.5	287.5	288.5	306.5	**260**	296.5	Random selection
Q5TAX3	TUT4_HUMAN	129	288	305	340	319.5	286.5	**278**	302	282	286.5	299	IsoSel
Q7Z429	LFG1_HUMAN	148	345	358	335.5	338.5	375	359.5	**333**	387	346	378	IsoSel

UniProtKB ID	Gene Name	Number of detected homologs	Tree lengths										Best(s) option(s)
			IsoSel parameters							Longest selection	Random selection	GUIDANCE	
			Default	-short	-gap	-GapShort	-DS	-DS -WOT	-auto				
O75390	CISY_HUMAN	158	281	278	**266.5**	284	299	276	298	280	297	286	IsoSel
P29275	AA2BR_HUMAN	168	353	353	**343**	344	359.5	357.5	**343**	353	357.5	353	IsoSel
Q96KR1	ZFR_HUMAN	134	362.5	318.5	270	323.5	367.5	332	288	327.5	**241.5**	331.5	Random selection
Q15417	CNN3_HUMAN	168	463	444.5	459.5	**444**	**444**	458.5	451	485	477	502.5	IsoSel
P29692	EF1D_HUMAN	148	399.5	389	397	412	390.5	401.5	398	**349**	361	408	Longest selection
P12955	PEPD_HUMAN	193	454	488	444	455.5	498	451.5	474.5	445	**435.5**	437.5	Random selection
O75317	UBP12_HUMAN	194	**517.5**	540	536	527	543.5	544.5	574.5	532	559	534.5	IsoSel
Q9H0J9	PAR12_HUMAN	165	398.5	**350**	373	362.5	397	386	368	359.5	404.5	394	IsoSel
P32322	P5CR1_HUMAN	178	506	520.5	510	474	479	483.5	479	**452.5**	486	486	Longest selection
Q5VST6	AB17B_HUMAN	203	447.5	**438.5**	461.5	492	472	468	490	462	476	442.5	IsoSel
O15127	SCAM2_HUMAN	170	369.5	338	375	323	326	321	372.5	340.5	**317.5**	352.5	Random selection
Q96ST3	SIN3A_HUMAN	172	273	259	262	275	261	288	272.5	266	**255**	282	Random selection
Q96FT7	ASIC4_HUMAN	168	442.5	431.5	399	437.5	452	429.5	429.5	397.5	**391**	445	Random selection
P04440	DPB1_HUMAN	129	315	321.5	313	**307**	313	330.5	317	317	315.5	324	IsoSel
Q8IVL1	NAV2_HUMAN	120	308	213.5	294.5	212.5	234.5	212	294.5	209.5	**193.5**	238.5	Random selection
Q9Y2D2	S35A3_HUMAN	213	727	755.5	663.5	**614.5**	666	710.5	711	701	764	707	IsoSel
Q6Q0C1	S2547_HUMAN	224	651.5	592	581	575.5	603.5	**573.5**	600	603	617	608.5	IsoSel
Q9UPU3	SORC3_HUMAN	219	314	318	304.5	348	320	293	310	335	319.5	**291.5**	GUIDANCE
Q9UPR5	NAC2_HUMAN	197	430.5	**384.5**	395	388	420	413	394.5	393	405.5	407.5	IsoSel
Q96S86	HPLN3_HUMAN	223	443	**430.5**	438	445	421	385.5	410	444	418	426	IsoSel
Q5JQF8	PAP1M_HUMAN	280	549	552.5	575	505.5	580	532	615	523.5	**470**	483.5	Random selection
P36268	GGT2_HUMAN	276	496	**450.5**	519.5	480.5	499.5	529.5	507.5	463.5	472	468	IsoSel
Q13367	AP3B2_HUMAN	287	519	**470.5**	523.5	503	501.5	569	571.5	500	494.5	489.5	IsoSel
Q08AH1	ACSM1_HUMAN	305	812.5	**656.5**	678	680.5	677.5	680.5	687.5	676.5	690	688.5	IsoSel
Q9Y2H2	SAC2_HUMAN	315	681	678.5	652	647.5	619.5	662	**616.5**	709	640	719	IsoSel
P16520	GBB3_HUMAN	350	865	870.5	856	830.5	833.5	846	854.5	**751**	883.5	859.5	Longest selection
Q9HCJ2	LRC4C_HUMAN	365	803.5	749	**740**	796.5	796.5	817.5	792	800	766.5	794	IsoSel
P20023	CR2_HUMAN	326	797	794	826	829	**780**	881	810	868.5	791.5	881	IsoSel
Q9HD20	AT131_HUMAN	407	721	**696.5**	742	743.5	781.5	726.5	714	726.5	720	700	IsoSel
P24928	RPB1_HUMAN	442	841	734	720.5	786.5	768.5	**717.5**	817	718.5	733	768	IsoSel
Q03052	PO3F1_HUMAN	349	1165	1147.5	**994**	1144	1139.5	1037.5	1096.5	1097	1057	1058	IsoSel
O95258	UCP5_HUMAN	440	1204	1205.5	1279	1142	1168	1208.5	1257.5	**1119.5**	1262	1149.5	Longest selection
Q6PIL6	KCIP4_HUMAN	402	1281	**1059**	1323.5	1334.5	1328	1288	1422.5	1341	1360.5	1367.5	IsoSel
P16190	1A33_HUMAN	316	568.5	551.5	531	528.5	547	**505**	543	526	578	572	IsoSel
P23760	PAX3_HUMAN	347	1286.5	1140.5	1178.5	1116.5	1255.5	1283	1274.5	**1003.5**	1284	1169	Longest selection
Q14952	KI2S3_HUMAN	325	**230**	490	490	495.5	495.5	495.5	495.5	495.5	269.5	490	IsoSel
O00338	ST1C2_HUMAN	556	1314	**1177.5**	1206	1267	1275.5	1288	1286.5	1228	1222.5	1219	IsoSel
P34998	CRFR1_HUMAN	446	940.5	952	964.5	965	923	937.5	918.5	**913**	934.5	983.5	Longest selection
P50995	ANX11_HUMAN	691	1171	1128.5	1193.5	1225.5	1157	1185	1167.5	1183	**1123**	1152.5	Random selection
Q8IW75	SPA12_HUMAN	614	1234.5	1210.5	1192	1160	**1159**	1182	1182.5	1184.5	1172	1210.5	IsoSel
Q13107	UBP4_HUMAN	576	1480	1393.5	1403	1442.5	1436	1545.5	1426	**1314**	1487.5	1541	Longest selection
P05121	PAI1_HUMAN	630	1353	1307	1294	1356	1361	1349.5	1419.5	1329.5	1392.5	**1290**	GUIDANCE
O00478	BT3A3_HUMAN	767	1588.5	1633.5	1658.5	1732	1590.5	1740.5	1761.5	1637	1633.5	**1566**	GUIDANCE
Q16352	AINX_HUMAN	791	1699	1744	1722.5	1760	1742.5	1763.5	1752	1758.5	**1692**	1759.5	Random selection
Q9UJA3	MCM8_HUMAN	968	2156	2071	2274	2336.5	2076	2170	2006.5	**1912.5**	2103	2154.5	Longest selection
Q99877	H2B1N_HUMAN	1043	3029	1471	2724.5	2682.5	1488	1648	1360	1341.5	**1200**	1419.5	Random selection
O00212	RHOD_HUMAN	1004	3286	3108	3396.5	3432.5	3064	**2991.5**	3089	3357	2996	3137	IsoSel
Q9P2N4	ATS9_HUMAN	807	1844	1901	1813	1770.5	1856.5	1850	1877	**1675**	1789.5	2026.5	Longest selection
Q02846	GUC2D_HUMAN	963	**2265**	2540	2495.5	2560.5	2340.5	2499	2275.5	2366.5	2454.5	2450	IsoSel
Q9NR20	DYRK4_HUMAN	1071	2735	2816.5	**2539**	3522.5	3298	3326.5	3318	3217.5	3337	3225.5	IsoSel
Q8NGZ2	O14K1_HUMAN	1285	2711	2736	2735	2736.5	2731.5	2736.5	2736.5	2751	**2686.5**	2721.5	Random selection
P31641	SC6A6_HUMAN	930	1838	2056	1995.5	**1734**	1841.5	1903	1773	1878.5	1840.5	1828	IsoSel
Q8N143	BCL6B_HUMAN	1173	4265.5	4415.5	4314	4653.5	4122	4627	4418.5	**4110.5**	4452	4294.5	Longest selection
Q00975	CAC1B_HUMAN	795	1831	**1699.5**	1792.5	1704.5	1847	1928	1906.5	1714	1805.5	1760.5	IsoSel
Q8NGT9	OR2A1_HUMAN	1484	3324.5	3282.5	3363	3291.5	3367	3367	3328	**3263.5**	3297.5	3323.5	Longest selection
O00743	PPP6_HUMAN	1352	3912.5	**3434.5**	3697	3843.5	3938	3720.5	4069.5	3949	3897.5	3661.5	IsoSel
P50993	AT1A2_HUMAN	1254	2512	2577.5	2622	2556.5	2568	2641.5	2544	2590	**2509**	2589	Random selection
Q8NH81	O10G6_HUMAN	1509	3392	**3305.5**	3341.5	3389	3356.5	3354.5	3348	3349.5	3308	3402.5	IsoSel
Q6ZN06	ZN813_HUMAN	1700	9428	9550	9818	8693	10080.5	8684	9550.5	**7540.5**	7852.5		Longest selection
P21506	ZNF10_HUMAN	1712	8071.5	8623.5	8692.5	7670.5	7620	8693	**7457**	8696.5	7804.5	8588	IsoSel
P58317	ZN121_HUMAN	1704	8370	8068	8314.5	8304.5	7526.5	7621	**7517.5**	7969	8460		IsoSel
Q08881	ITK_HUMAN	1494	4383	4403	4309	4416.5	3991	4080.5	3951	3936.5	**3933**	4272.5	Random selection
P09769	FGR_HUMAN	1507	4495	4523	4828	**4309**	4625.5	4732.5	4820.5	4451	4421	4404.5	IsoSel
Q8N8J6	ZN615_HUMAN	1770	**7665.5**	9483	9109.5	8624.5	8907.5	8620	9306	9474	9009		IsoSel
Q16816	PHKG1_HUMAN	1490	3458	**3105.5**	3221.5	3405	3301.5	3468.5	3239	3331	3410	3392.5	IsoSel
Q7L7X3	TAOK1_HUMAN	1472	4798	5215	4563	5113	4787	5439.5	4884	4468.5	**4188**	4456.5	Random selection
Q9Y243	AKT3_HUMAN	1564	3543.5	**3320**	3365.5	3809.5	3489	3436	4205.5	3492	3683.5	3428	IsoSel
		**Means :**	**907.17**	**901.062**	**917.58**	**910.338**	**897.09**	**907.71**	**905.61**	**881.40**	**882.27**	**744.88**	
