# Supplementary figures and images for: IsoSel: Protein Isoform Selector for phylogenetic reconstructions
Source: PLoS One. 2017 Mar 21;12(3):e0174250. doi: 10.1371/journal.pone.0174250 (PMC5360266; doi:10.1371/journal.pone.0174250)

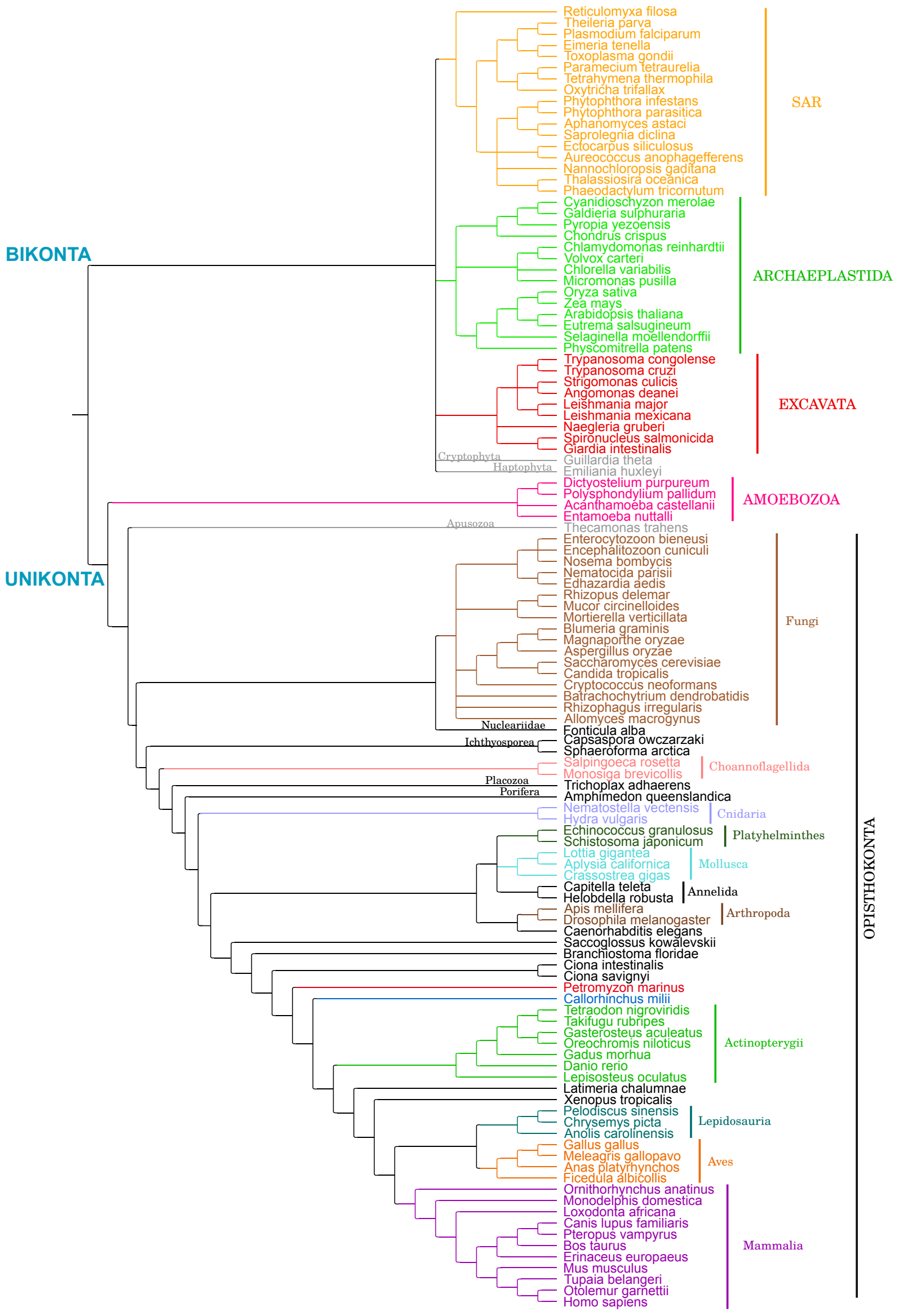

Supplement: S1 Fig — This tree was built according to Lecointre and Le Guyader book [21], the Ensembl reference species tree for the metazoan part [22] and a personal communication from CBA. (PDF) [file pone.0174250.s006.pdf]
